# A Historical Review of the Artificial Pollination of *Vanilla planifolia*: The Importance of Collaborative Research in a Changing World

**DOI:** 10.3390/plants13223203

**Published:** 2024-11-15

**Authors:** Adam P. Karremans

**Affiliations:** Lankester Botanical Garden (JBL), University of Costa Rica (UCR), Cartago P.O. Box 302-7050, Costa Rica; adam.karremans@ucr.ac.cr

**Keywords:** climate change, Charles Morren, Edmond Albius, genetic diversity, Middle America

## Abstract

The natural fertilization of *Vanilla planifolia* has long been a matter of speculation. Stingless (tribe Meliponini) and orchid bees (tribe Euglossini) are often cited as effective pollinators, but direct evidence is notoriously lacking. As with other food-deceptive orchids, the natural fruit-set in *V. planifolia* is low and does not occur spontaneously outside its native range in Middle America. Fruiting has, therefore, necessitated human intervention through artificial pollination. How *Vanilla* first came to be artificially pollinated is a controversial issue spurring egotism and nationalism. There are numerous texts offering differing versions of the historical events that led to the discovery of the artificial fertilization of *V. planifolia* and its propagation as a crop. Historical records show *Vanilla* was simultaneously being pollinated in several parts of the world. I argue that the suspected independent simultaneous discoveries made in Liège, Paris, Padua, the Dutch colony of Java (Indonesia), and the French insular colonies Réunion (Bourbon), Guadeloupe, and Martinique are not unconnected. I conclude that they can be traced back to a single discoverer whose feat was spread around the globe by a tight network of corresponding naturalists. This view contrasts with previous authors. Finally, current concerns regarding *Vanilla* pollination and production are addressed, highlighting the need for immediate actions to conserve the genetic diversity of the crop’s wild relatives to attenuate the effect of extreme climates in a changing world. A plea is made to shift the focus to Middle America, stimulating and supporting local research and conservation efforts and the allocation of funds from this billion-dollar industry.

## 1. Introduction

Vanilla is a vital ingredient for many industries worldwide and has become an important cash crop for smallholder farmers and local economies around the tropics. The world market for this spice, which is naturally obtained from the fruit of the *Vanilla* Plum. ex Mill. plant, is estimated at USD 1.26 billion [1]. However, the vanilla crop faces important threats due to significant genetic erosion and vulnerability of the crop’s wild relatives [2]. FAO data suggest the world production of vanilla was about 8000 tonnes per year between 2010 and 2019 [3], with the main producers being Madagascar (3033 tons/yr), Indonesia (1965 tons/yr), Mexico (710 tons/yr), Papua New Guinea (491 tons/yr), and China (431 tons/yr), according to the FAOSTAT 2022 [1].

Over 90% of vanilla is produced in countries that are outside the natural range of the crop and its wild relatives. *Vanilla planifolia* Andrews, the main crop species, is native to Middle America. Outside of the neotropics, vanilla plants do not fruit on their own due to the lack of natural pollinators [4,5]. In vanilla plantations, flowers of *V. planifolia* are, therefore, mainly pollinated artificially. The lack of natural pollinators in the major vanilla-producing countries forces farmers to rely on hand pollination alone, a very labor-intensive and delicate activity [6]. As hand pollination results in higher pollination rates and thus yields compared to natural pollination, this technique has been applied in all commercial vanilla plantations worldwide, including those in neotropical countries [7].

Little is known about the natural pollination and phenology of species belonging to the orchid genus *Vanilla* in their natural habitat [8]. *Vanilla* species are mostly pollinated by means of autonomous self-pollination or animal vectors [9,10,11,12,13], yet few studies provide undisputed evidence on pollination events. Pollination by Euglossini has been confirmed for the close relatives of *Vanilla planifolia*, and these so-called orchid bees are suggested as the effective pollinators of most Neotropical *Vanilla* species (e.g., [8,9,11,13,14,15,16,17]), by means that include food-deception, nectar rewards, or fragrance rewards [8,13]. Given the scarcity of evidence of the natural pollination of *V. planifolia* itself, despite the publication of several works attempting to address the issue (see review below), the actual pollinators and mechanisms are still debated. However, studies agree that even when bees are present, natural fertilization is rather infrequent and thus unreliable for commercial production.

Up until the eighteenth century, vanilla spice came exclusively from tropical America, where wild vanilla was gathered rather than cultivated [18]. By the 1760s, the Papantla region in Veracruz, Mexico, emerged as the first in the world to cultivate and produce vanilla for international export. Its sole destination was the European market [19]. The discovery of how to pollinate vanilla artificially led to what is known as the ‘*Vanilla* revolution’ in the mid-nineteenth century by allowing its cultivation in the Old World tropical colonies [18]. But the details of how the vanilla revolution began is a controversial issue that has spurred egotism, nationalism, and cultural pride. There are numerous texts, both formal and informal, offering versions of the historical events that took place, especially those that led up to the discovery of the artificial fertilization of *Vanilla* and its propagation as a commercial crop around the world. Most of this literature, especially in modern times, credits Edmond Albius, a creole from Réunion island (Bourbon), as the “true and only” discoverer of the artificial pollination of *Vanilla*. Yet, I will argue that this is an overstatement, a modern claim never made by the persons involved and based on the misunderstanding of historical facts. Albius certainly played a crucial role in figuring out and implementing a practical way of pollinating *Vanilla* flowers, consequently turning it into an important crop in Réunion and later the whole region. Yet, he was neither the first to discover how to fertilize vanilla flowers nor can he be credited as the sole figure expanding vanilla culture worldwide, effectively igniting the *Vanilla* revolution of the nineteen hundreds.

Arditti [20] noted that there were clear historical records that *Vanilla* was being pollinated in several parts of the world around the time of Edmond’s famous discovery, allegedly, in 1841. The author suggested these events were probably unconnected and hypothesized multiple independent discoveries of the process of *Vanilla* pollination [20]. Rather, based on the available evidence and historical circumstances, I will argue that the suspected simultaneous, yet independent, discoveries made in Liège, Paris, Padua (Padova), the island of Java (Dutch colony of Indonesia), and the French colonies Réunion (Bourbon), Guadeloupe, and Martinique are not independent at all. I conclude that the most parsimonious explanation is that they are all connected and can be traced back to a single discoverer: the Belgian naturalist Charles Morren. Given that this view contrasts with virtually all previous authors who have written on the subject, I invite the reader to follow me through the timeline of historical events and draw their own conclusions.

Hereafter, I first address the natural pollination of *Vanilla planifolia* and why it has been necessary to “discover” an artificial method of fertilizing its flowers. Then, I argue that the understanding of the fertilization of orchid flowers and the spread of *V. planifolia* plants led Charles Morren to discover the artificial pollination in *Vanilla*. This is followed by an account concerning the spread and global implementation of his teachings thanks to a tightly knit community of naturalists. Finally, I address *Vanilla* culture in a changing world, stressing concerns regarding pollination and production, and the conservation of the genetic diversity of the crop and its wild relatives.

## 2. The Unreliable Natural Fruit-Set of *V. planifolia*

*Vanilla* flowers mainly rely on animal vectors for pollination and thus require pollinator visitation in order for them to fertilize naturally. Pollination strategies among *Vanilla* species are varied. These include (1) food deception, meaning the flowers signal rewards that are not present; (2) floral rewards, meaning the flowers offer food in the form of nectar; or (3) a dual mechanism in which the flower offers resources in the form of chemical fragrances, but also deceives the pollinator by signaling, yet not offering, food. Neotropical *Vanilla* species, and in particular those species with aromatic fruits, have been hypothesized to be mainly pollinated by Euglossine bees by Soto Arenas [21]. These bees are known as orchid bees for their crucial role in the reproduction of numerous orchids in the Neotropics. A review of the literature shows that *Vanilla* pollinators have been found to be mainly bees in the Apidae family, and indeed, notwithstanding the gaps in our knowledge, the aromatic species from Middle and South America are shown to mostly require the intervention of orchid bees to become fertilized [8].

The details behind the natural fertilization of *V. planifolia* specifically have long been a matter of speculation, with direct evidence still notoriously scarce. Stingless bees (Meliponini) are often cited as the suspected pollinators of *V. planifolia* in the literature, going as far back as the nineteenth century. Even though this notion is repeated over and over, there is actually no evidence for it. I find that the origin is most likely Delteil [22], who in 1884 stated that in French Guiana and Mexico, small bees carried out the fertilization while collecting pollen and nectar from the flower. According to Delteil, the insects, which belonged to the genus “mélipone”, were of a number of varieties, “varying in color, pale green, striped yellow, chestnut, brown, black and even blue” with sizes ranging “1 to 7 cm”. The author noted, “Hummingbirds also flutter around the vanilla flowers and insert their beaks into the sexual organs of the flowers, thus causing fertilization *”.

Arthur Delteil visited French Guiana in 1871, and it is likely that these observations on floral visitors were made during the trip. His comments have been translated and copied over decades and eventually were taken very literally to mean ‘*Vanilla planifolia* is pollinated by Melipona bees’. But Delteil speaks of bees that are of pale green, striped yellow, chestnut, brown, black, and blue colors. He also talks of these insects being between 1 and 7 cm in length, which is unheard of among bees, let alone Meliponini. At the time, neotropical bees were not as well known as they are today, and his comments suggest he was mingling different species from diverse genera of bees. Perhaps both Euglossini (orchid bees) and Meliponini (stingless bees), but possibly also other insects. Orchid bees of the genus *Euglossa* are, as mentioned by Delteil, often pale green, chestnut, or blue colored, while those belonging to the genus *Eulaema* are typically black with yellow bands or stripes. Most importantly, although several *Vanilla* species are native to French Guiana, *V. planifolia* is not, nor does its flowers offer pollen or nectar to the visiting bees. Therefore, the observations made by Delteil are most likely based on a mixture of different *Vanilla* species and visiting animals and cannot be taken in the strict interpretation it has become after numerous rewritings.

**Figure 1:**
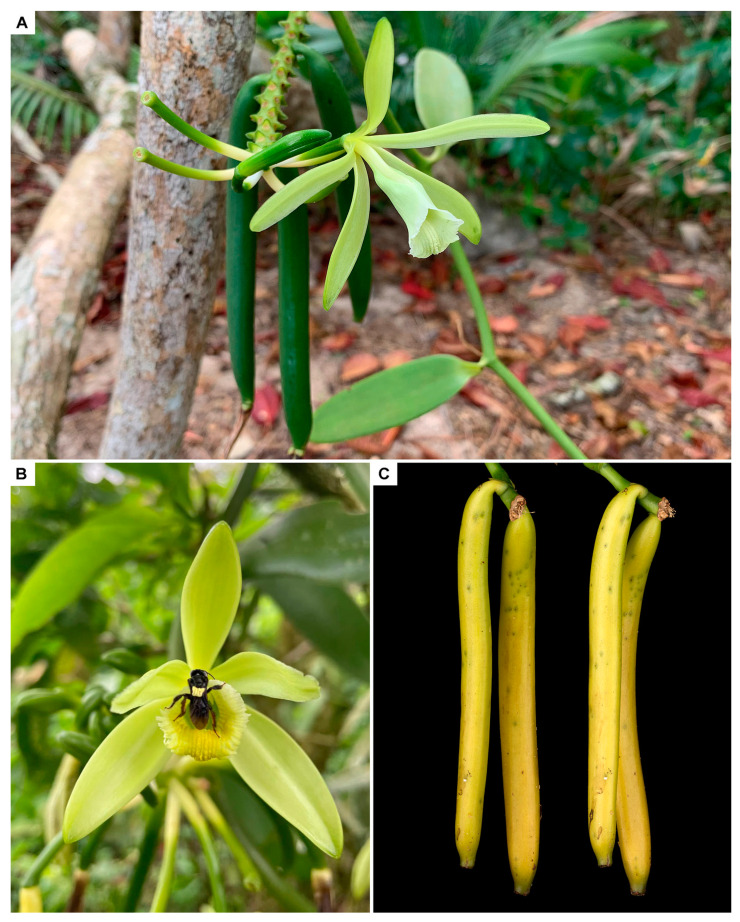
*Vanilla planifolia*. (**A**) Natural flowering and fruiting in a wild population in Cahuita, Costa Rica. (**B**) A stingless bee removes pollen grains on its back while exiting a flower in San Carlos, Costa Rica. (**C**) Naturally set fruits are proportionally rare in nature. Photographs by the author (**A**,**C**) and Randy Domínguez Miranda (**B**).

A study by Ackerman [14] on Barro Colorado Island in Panama evidenced the presence of pollinia of ‘*Vanilla fragrans*’ on the scutellum of nine male individuals of *Euglossa tridentata* bees. The name *V. fragrans* is an invalid taxon often considered a synonym of *V. planifolia*—although, formally, it is not [5]. Based on common usage, one might be tempted to assume that Ackerman referred to *V. planifolia*. However, the name *V. fragrans* has also been used for several other closely related taxa, and the taxonomy of the Central American *Vanilla* was not thoroughly revised until decades later [16]. Eight *Vanilla* species are known to occur in Panama, at least half of them in the Barro Colorado area [5,23]. This includes *Vanilla hartii*—a small-sized species easily confused with *V. planifolia*. Extensive sampling of Euglossini within the large *V. planifolia* populations in Cahuita (Figure 1A), Costa Rica, has yet to produce a single male bee carrying its pollinaria [24,25,26]. In the first, sampling was carried out at random times using only mature *V. planifolia* fruits as bait. However, the second included several baits and systematic sampling throughout the year, fully covering the *V. planifolia* flowering period. *Euglossa tridentata*—the bee collected with *Vanilla* pollinaria in the Ackerman study—was one of the most commonly captured male bees in both cited studies, and yet it never carried any *V. planifolia* pollinaria despite the plants profusely blooming and being overwhelmingly abundant in the area. On the contrary, a study by Watteyn et al. [8] showed that both males and females of *E. tridentata*, among others, were the most likely pollinators of *V. hartii* in southern Costa Rica. We cannot definitively rule out that *V. planifolia* was indeed pollinated by male Euglossine bees on Barro Colorado. But there is good reason to suspect that perhaps the pollinia found by Ackerman [14] belonged to a different species—such as *V. hartii*—instead.

In Mexico, Soto Arenas [16,21] suggested that *Euglossa viridissima* pollinated the flowers of *V. planifolia* through a deceptive system possibly involving floral fragrances. However, no further evidence of pollination by *E. viridissima* was given. During a two-week period in the spring of 2004, Lubinsky et al. [9] observed occasional visits by ants, bees, and hummingbirds, but no pollination. Visiting insects reported by the authors included species belonging to the orchid bee genera *Euglossa* and *Exerete* and the stingless bee genus *Melipona*. Stingless bees were also observed by Quezada-Euán et al. [27] when visiting the flowers of *V. planifolia* in Mexico without being effective pollinators. We have observed ants and stingless bees being frequent floral visitors of *Vanilla* species in Costa Rica, including *V. planifolia*. They often step onto and inspect the flowers while dutifully patrolling the inflorescences and ovaries, searching and consuming the abundant, sweet extrafloral nectar that many *Vanilla* offer. They surely can remove pollinaria on occasion (Figure 1B). However, I agree with Dressler [28] and Lubinsky et al. [9] that it is unlikely that they are the main pollinators, given their small size. A study by Pemberton et al. [29] carried out in a commercial nursery in Broward County in southeastern subtropical Florida found a single female of *Euglossa dilemma* carrying pollinaria of *V. planifolia*. Unfortunately, neither *V. planifolia* nor *E. dilemma* are native to Florida, but their study provides important evidence for what one may expect to occur in their native regions.

Be it as it may, the natural fruit-set of *V. planifolia* is extremely low, consistent with what is often observed in food-deceptive orchids [30]. Soto Arenas and Dressler [16] suggest between one in a hundred and one in a thousand flowers of *V. planifolia* are pollinated naturally in Mexico. This is consistent with the 0.655% fertilization ratio that was found in southern Florida [29], and the 4.9% found in Yucatán, Mexico [27], as well as with our own ongoing studies on the Caribbean coast of Costa Rica (Figure 1C), in which one fruit is observed per every few hundred flowers [26]. Pemberton [31] suggests that non-perfume orchids, including some *Vanilla* species, can be pollinated by female and, to a lesser extent, male orchid bees searching for nectar. A strategy involving euglossine females and food deception, rather than perfume-collecting males, explains why *V. planifolia* produces so many flowers, and yet it is so rare to observe fructification and, consequently, pollination occurring naturally. It would also explain why, despite the numerous male baiting efforts carried out in Mexico and Central America, *V. planifolia* pollinaria are not found on any of the bees caught. Despite being related to honeybees and stingless bees, orchid bees are solitary, they do not live in large, communal colonies and, therefore, are not easily cultured. This, in addition to the flower relying on food deception, makes prompting natural pollination under cultivation by artificially supplying pollinators rather challenging.

## 3. Understanding the Fertilization of Orchid Flowers

The adaptation of the flowers of *V. planifolia* to a pollination mechanism based on the deception of solitary bees explains why the fruit-set is naturally low, even when the natural pollinators are present. In geographical areas where these insects are unavailable, the fruit-set is even more unlikely, although it has been reported to occur occasionally. The bee genera *Melipona* and *Trigona*, as well as the whole tribe Euglossini, are endemic to the tropical areas of the Americas. These currently confirmed and suspected natural pollinators of *V. planifolia* are all absent from Africa and Asia. Therefore, it is no surprise that when this tropical vine was sent to be cultivated in the European colonies in the New World, it grew well but never produced any fruits. It took farmers a while to figure out why this was.

*Vanilla* fruits were being collected in the American tropics, sold, and exported to Europe for centuries before the first records of artificial pollination were made in the 19th century. Vanilla was a popular spice used in chocolate beverages in Europe in the 17th century, and there are several records of vanilla trade from that time [18]. For example, Jewish settlers secured the chocolate trade in Jamaica in 1655 by figuring out how to cure vanilla. The pirate Dampier describes Indians selling vanilla to the Spanish in Bocas del Toro in Panama in 1660, while another record describes its presence in the Bay of Campeche and the coast of Veracruz in Mexico in 1676 [32]. These observational texts speak of *Vanilla* fruits being solely collected from naturally pollinated wild plants, and there are no available records that would suggest that flowers were being hand-pollinated by natives.

Current available records show that it was not until the end of the eighteenth and early nineteenth centuries that naturalists began to understand the purpose and function of floral organs in plants. Before that, early botanists had often concentrated their efforts on describing the morphological features of plants, classifying them in order to establish their potential use as foods, medicines, or tools. Ecological interactions were considered a mere intellectual curiosity rather than an academic priority [33]. The notion that flowers require and actively employ strategies to become pollinated by means of animal vectors was only postulated in the year 1793 when Sprengel published the findings of his groundbreaking observations in *Das Entdeckte Geheimnis der Natur im Bau und der Befruchtung der Blumen* [Discovery of the Secret of Nature in the Structure and Fertilization of Flowers]. Among his most important postulations, Sprengel [34] asserts that pollen masses need to be directly applied to the viscid surface of a stigma for flowers to become fertilized and points out insects are the main agents behind this process.

This general trend in flowering plants also applies to orchids. It is only after these initial works by Sprengel that we find the first record of any orchid flower being artificially pollinated. German botanist J.K. Wachter [35] published his results on the hand pollination of *Platanthera bifolia* (L.) Rich, probably carried out in the year 1798 [36]. The feat was repeated by Salisbury [37], who asserted that he had also been successful in artificially fertilizing several orchid species by applying the pollen masses directly to the stigma. Curiously, between the end of the 1700s and the first quarter of the 1800s, researchers were still actively debating whether the direct application of pollen to the stigma was, in fact, necessary for orchids to become fertilized. Given the peculiar structure of flowers, certain botanists considered it too difficult for pollen to reach the stigma externally and, therefore, unlikely that this would be required for fertilization to occur at all. Linnaeus himself wondered if orchid pollen could be transferred internally to the ovarium without requiring any external intervention or displacement, a view that was shared by several other authors. The matter was not settled until Robert Brown read a thorough review of the mode of fecundation of orchid flowers in November of 1831 before the Linnean Society of London. In his essay, Brown [38] described the function of the different organs that compose the orchid flower and offered a clear explanation of how orchid flowers become fertilized.

Key observations on the reproduction of orchids were offered also by Brongniart [39], who first read the work *Observations sur le mode de fécondation des Orchidées et des Cistinées* before the Royal Academy of Science of Paris on the 4th of July 1831. Adolphe Brongniart was one of the botany professors of Charles Morren while studying in Paris [40], and they corresponded on the fertilization of *Vanilla,* as we will address further below [41].

## 4. The Vine That Conquered the World

While the intricate details behind the fertilization of orchid flowers were beginning to be unraveled, another key circumstance for the discovery of self-pollination in *Vanilla* was developing in Europe.

**Figure 2:**
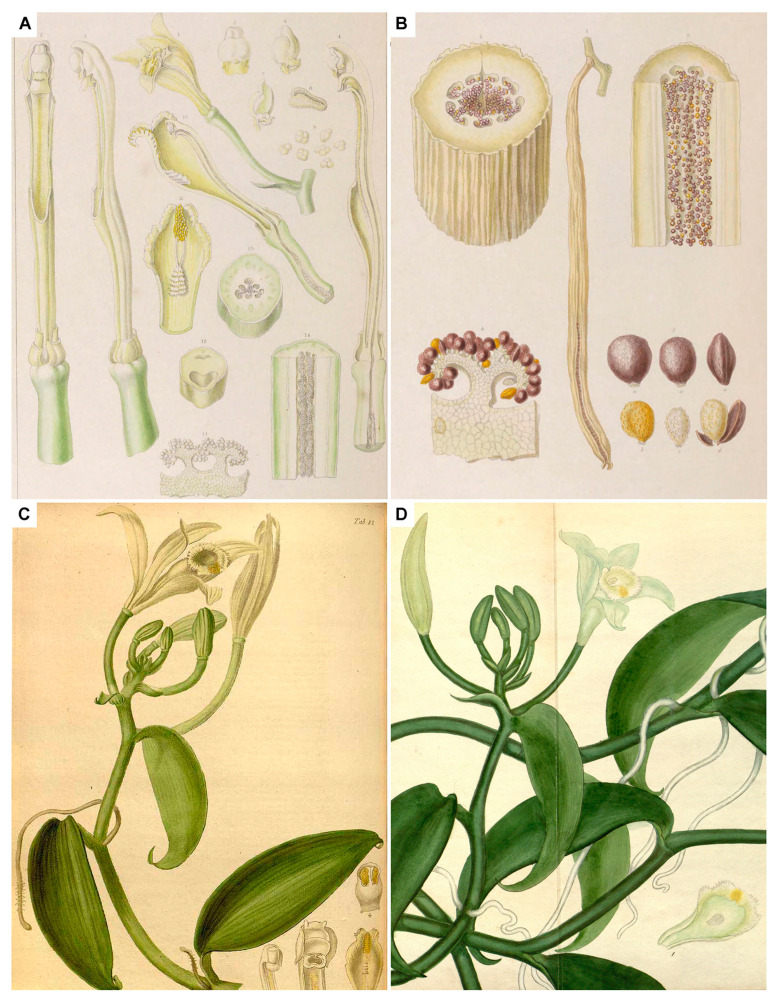
Original illustrations of *V. planifolia* from Greville’s collection. (**A**,**B**) Detailed sketches were prepared by Franz Bauer and published in the *Illustrations of orchidaceous plants*. (**C**) Illustration by Sir William Jackson Hooker published in the *Paradisus Londinensis* accompanying the description of the botanical name *Myrobroma fragrans* Salisb. (**D**) Nomenclatural type for the name *V. planifolia* Andrews published in the *Botanist’s Repository*. ©Biodiversity Heritage Library.

In the year 1800, a *Vanilla* plant was introduced by George Spencer Churchill, the Marquis of Blandford, who would later become the fifth Duke of Marlborough, to his private collection in Whiteknights, in Reading, England [18,42]. From the collection of the Marquis, cuttings of the plant are spread to several collections and make it to the estate of Right Hon. Charles Greville at Paddington, where they thrive and bloom for the first time in 1807 [42,43]. There are three known illustrations of the plant that flowered in Mr. Greville’s collection. Detailed sketches were prepared by famous botanical illustrator Franz Bauer (Figure 2A,B), and were published together with Lindley in 1834 [44]. Another illustration was prepared by his pupil Sir William Jackson Hooker, a botanic painter to their majesties at Kew. It was published in *The Paradisus Londinensis* [43] and accompanied the description of the botanical name *Myrobroma fragrans* Salisb. (Figure 2C). A third, prepared by Andrews, was published in the *Botanist’s Repository* [45] and served as a nomenclatural type for the name *Vanilla planifolia* (Figure 2D). Contrary to common belief, the origin of the plant cannot be traced to Mexico, and the exact provenance remains unknown. The original description of *V. planifolia* mentions only the West Indies [45], and given that the Marquis was known to import plants from Jamaica [46], this country has been suggested as a possible origin for the *Vanilla* [18]. We cannot say for sure if the *Vanilla* plants in the collection of the Marquis had been imported from Jamaica, but we know today that *V. planifolia* is not native to the West Indies [5], so they must have originated elsewhere.

In 1812, cuttings of *Vanilla planifolia* from Mr. Greville were introduced into cultivation by Joseph Parmentier, mayor of Enghien (Belgium), and entrusted to the care of Claude Louis Sommé in the botanical garden of Antwerp [42,47]. The plant thrived under the care of Dr. Sommé in Antwerp, and he took it upon himself to send it out to “all” Botanical Gardens and collections across Belgium and France [42]. By 1814, Sommé confirmed that the *V. planifolia* of English origin grew well in the greenhouses in Antwerp but had not flowered anywhere in Belgium except at Ms. Viscount Vilain XIIII’s collection in Wetteren [47]. It was later confirmed to grow and bloom in the Belgian cities of Ghent (Gand) in 1818 and Liège in 1829, both at that time part of the United Kingdom of the Netherlands.

*Vanilla planifolia* grew well in the greenhouses of Europe and, from there, was sent to the tropical colonies of France and The Netherlands. Sommé was responsible for providing Joseph Marchal, a former employee of the Dutch East Indies from Brussels, with five potted *Vanilla planifolia* cuttings to take from Antwerp to Batavia (Java) for acclimatization in the Dutch Indies in 1819 [42,47]. A year later, a single cutting of *V. planifolia* taken by Marchal survived the 181-day journey to Java (Indonesia) and was cultivated with difficulty at Buitenzorg. In 1840 or 1841, C.G.C. Reinwardt and Carl Ludwig Blume, director of the national herbarium of The Netherlands (Rijksherbarium), recommended the Dutch government to transfer *V. planifolia* and make a new attempt to introduce *Vanilla* culture into Java [48,49]. About two dozen plants were purchased from Belgium and brought to Leiden, from where Mr. Pierot would take them to Java for cultivation, constituting the second introduction of the species to Indonesia [50]. In a letter dated 16th December 1844, Teijsmann [51] confirms that the *V. planifolia* plants sent to Java were growing well, and had now bloomed. De Vriese [49] also tells us that plants from Leiden were sent to Suriname.

In 1822, Marchant, the previous colonial administrator on Réunion island (*ordonnateur de l’île Bourbon*), visited L.A.G. Bosc in Paris and obtained *V. planifolia* cuttings to be cultivated at the estate of his mother-in-law Madame Fréon at la Belle-Eau, Réunion [52]. It is believed that it is the descendants of these plants that Féréol Bellier-Beaumont cultivated at his estate in Bellevue, Sainte Suzanne. In 1833, a *V. planifolia* plant flowered in the Botanical Garden of Padua (today Italy, but at that time under Austrian rule), where it flowered again in 1840 and 1841, while mature fruits first dropped in May of 1842 [53].

## 5. Charles Morren and the Discovery of Artificial Pollination in *Vanilla*

**Figure 3:**
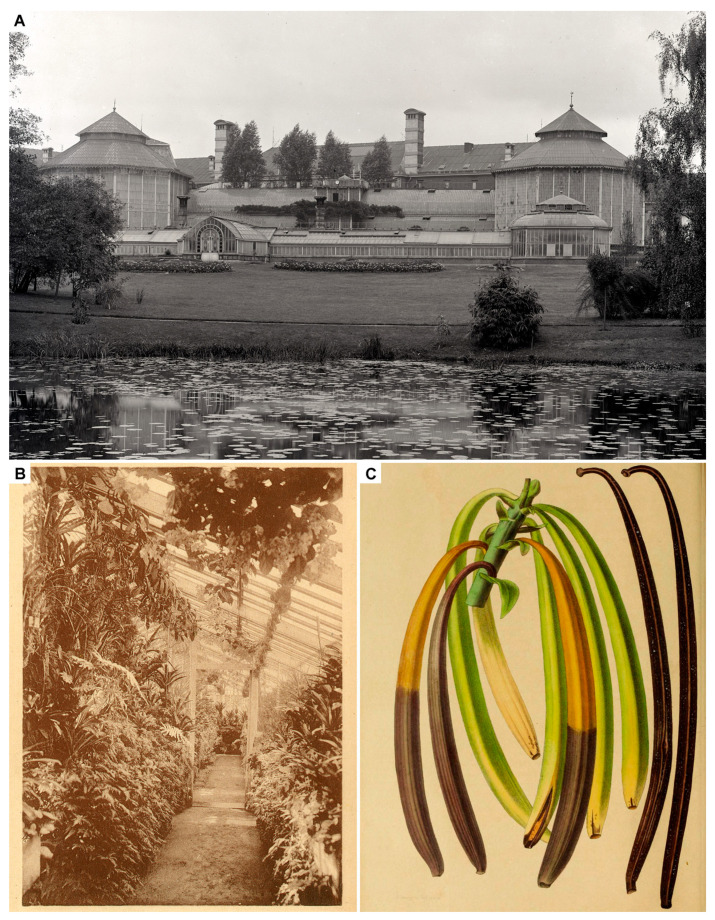
(**A**) View of the greenhouses of the Botanical Garden of Liège ca. 1880–1890, before their partial destruction during WWII. (**B**) Postcard from ca. 1920–1930 showing *V. planifolia* on the top-left growing at the botanical garden. (**C**) Fruits of *V. planifolia* obtained by C. Morren, possibly illustrated by E. Morren. Photographs by Victor Barras (**A**), and unknown (**B**). ©Province de Liège—Musée de la Vie Wallonne.

Charles François Antoine Morren, born in Ghent on the 3rd March 1807, was a Belgian naturalist with a predilection for botany, horticulture, and paleontology. He occupied the botany chair at the Université de Liège and, after the untimely passing of his predecessors Henri Gaëde and Richard Courtois, became director of the *Jardin Botanique* in 1835 (Figure 3A). Being a true naturalist, Morren delved into fields as diverse as plant physiology, comparative anatomy, descriptive botany, paleontology, geology, zoology, and medicine. Besides being an extraordinary scholar, professor, and orator, he was also an illustrator and a poet [40].

At Liège, Morren experimented on many tropical plants, often presenting his results before the numerous botanical, horticultural, and scientific societies of which he was a member. Morren wrote on plant movement and excitability, anatomy, morphology, and development. Morren had a special interest in orchids, describing 30 or so species, working on the anatomy of *Orchis latifolia* L. [40], and experimenting with the fertilization of *Leptotes bicolor* Lindl. [54]. At Liège, he experimented on *V. planifolia*, a plant that grew profusely in the greenhouse (Figure 3B) and flowered yearly, according to Courtois, who had first prepared a specimen in 1829 [52]. Interestingly, Morren states that his experiments on the fertilization of *Vanilla* are informed by the detailed sketches of the sexual organs of *V. planifolia* made by Franz Bauer in 1807 and the 1831 works by Robert Brown and Adolphe Brongniart on the fertilization of orchids [41]. On the 16th of February 1836, Morren became the first person on record to artificially fertilize a *Vanilla* flower, something he double-checked with correspondents, including Brongniart, de Mirbel, and others [41]. Morren successfully produced 54 pods by self-pollinating flowers of *V. planifolia* cultivated at the Botanical Garden (Figure 3C). One year later, in February of 1837, the first pod fully matured in Liège [41,55,56]. From a second plant, he obtained 100 fruits.

His successful artificial fertilization method is described as follows: “The column presents, above the stigmatic surface, a duplication in the form of a fleshy apron, which descends in front of the stigma and separates it from the operculum, in which the pollen masses are contained… Upon lifting or removing the apron, the entire pollen mass, or just a part, is brought into contact with the stigma… many flowers can be fertilized *”. Morren [55]. “From this structure it results, that all approximation of the sexes in this orchideous plant is naturally impossible. It is thus necessary either to raise the velamen or to cut it when the plant is to be fecundated, and to place in direct contact the pollen and the stigmatic surface… If impregnation has been effected, the petals and sepals reverse inwardly, and the flower droops instead of remaining erect. So soon as the following day the ovarium elongates” [56]. Morren displayed a branch of ripe fruits at the meeting of the horticulture society of Ghent at the Casino on 10th March 1837. The Grand Jury wanted a special medal, on which this memorable success would be engraved, to be awarded to Morren. He was obsessed with Vanilla and had a special greenhouse built near his home on rue Louvrex in order to cultivate and make the vanilla plant bear fruit on a larger scale. He would have his wife, Marie Morren (née Marie Verrasselt), present the *Vanilla* fruits at the horticulture exhibit in Namur to circumvent the rule prohibiting a member of the jury from participating in the contest [52].

Besides securing Prof. Morren a silver medal, the demonstration in Ghent also got him an invitation from members of the Royal Horticulture Society of Paris to publish his work. In their report of the meeting of the horticulture society, vice-president Lorenzo Berlèse and rapporteur Pierre Antoine Poiteau commented: “If Morren does not publish his process and his results soon, we will have the honor of communicating to you what he was kind enough to tell us about it himself *” [57]. In their follow-up report on the 10th March 1837, Poiteau and Berlèse, reiterate: “We will not finish this report, gentlemen, without reminding you once again of the *Vanilla* fruit presented at this exhibition by the learned professor at the University of Liège, Morren. This fact, new for us and for the whole of Europe, deserves the greatest publicity *” [58]. Their desire was promptly satisfied, and on the 5th of April 1837, when Mr. Berlèse had the honor of reading before the members of the Royal Horticulture Society of Paris news that Mr. Morren had successfully obtained fruits of *Vanilla* at Liège [55]. The council voted to thank the author and sent the notice to the editorial committee for publication. Morren’s paper on the pollination of *Vanilla* was finally published in May of 1837.

“The University botanical garden contains very beautiful plants; but what caused me the most pleasant surprise was to see a *Vanilla* plant loaded with around thirty fruits in perfect condition, and whose ripeness was not far off *” The presence of *Vanilla* fruits at the botanical garden of Liège was independently confirmed by a mister Camuzet, of the Garden du Roi, another member of the Royal Horticulture Society of Paris, who in September of 1837 visited Liège while on a horticulture tour of Belgium [59]. On the 3rd of June 1838, French botanist Étienne Soulange Bodin, general secretary of the Royal Horticulture Society of Paris, noted that the 2.5 kg of *Vanilla* that had been produced at the University of Liège was an accomplishment that could one day find a very lucrative branch of the horticultural industry [60].

Morren’s work was never meant to be restricted to a scholarly audience, as has been unfortunately stated (see below). He presented the results of his work to numerous botanical and horticultural societies, clearly expressing that the technique deserved commercial attention as it could provide a great abundance of fruit in the colonies. Upon presenting his discovery in Newcastle, Morren [56] informs: “in all the intertropical colonies vanilla might be cultivated and a great abundance of fruit obtained by the process of artificial fecundation… It is a subject which well deserves attention in a commercial point of view; and is moreover a proof of the importance of science for improving every branch of industry”. Perhaps most importantly, Morren suggested that the *Vanilla* fruits produced at Liège could rival those from Mexico, and proposed expanding his trails into commercially productive plantations. He was never given permission by the Belgian government and had to desist given that the law on higher education prohibited professors from exercising another profession unless extended special authorization from the authorities [52]. Morren lamented the lack of enthusiasm of the Belgian authorities but was aware that based on his teachings, the artificial pollination of *V. planifolia* had been successfully repeated in Ghent, Paris, London, Hamburg, and Padua [48].

**Figure 4:**
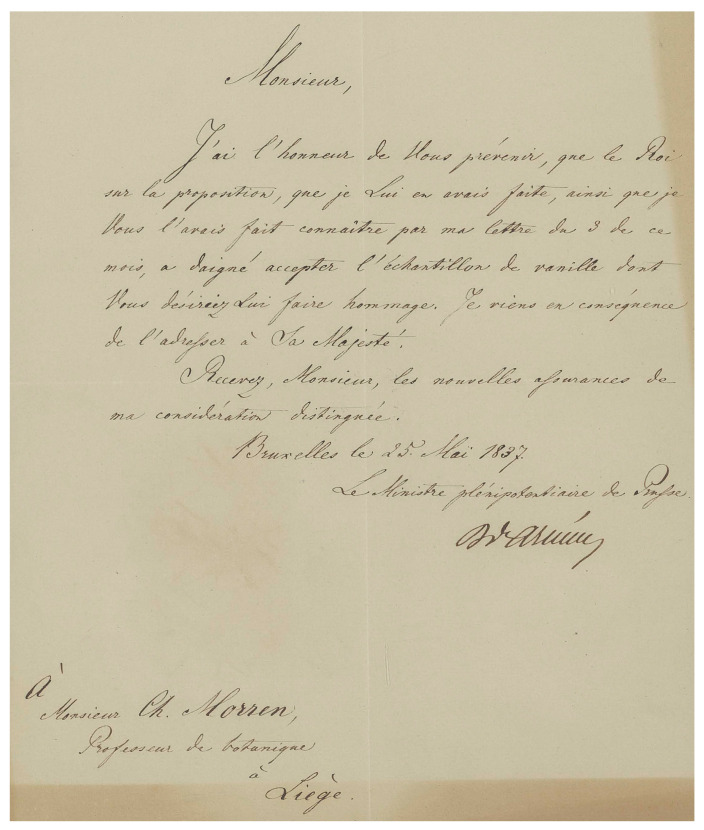
A letter by the Prussian plenipotentiary diplomat to Brussels dated 25th May 1837, reads: “I have the honor to inform you that the King, on the proposal that I made to him, as I had made known to you by my letter of the 3rd of this month, dignified to accept the sample of vanilla with which you wish to pay him tribute. I have therefore just addressed it to His Majesty *”. Photograph by Laurent Gohy, reproduced with the kind permission of the Central Library of the University of Liège.

Morren profusely traveled across Europe, visiting Britain, France, Germany, Italy, Luxembourg, Sweden, Switzerland, and The Netherlands. Between 1830 and 1852, he was named a corresponding or full member of 36 regional or national academies and societies of natural sciences [40]. Morren consequently had an extensive network of important correspondents. This includes famous French naturalists such as Brongniart, de Candolle, Cuvier, Delessert, Gaudichaud-Beaupré, de Jussieu, de Mirbel, Redouté, Seringe, the German botanist Link, Italian botanists Brignoli of Brunhoff, Moris, Passerini, Tenore, and British botanists John Lindley, Robert Brown and Sir William Hooker, among many others. His agricultural, botanical, and horticultural works gave him significant notoriety. As early as May of 1837, the Prussian King accepted a sample of his *Vanilla* fruits presented by Morren (Figure 4).

Morren became so influential that he was conferred knighthood status on multiple occasions. Official condecorations for which I could find records include Knight of the North Star of Sweden and Norway in 1846; Knight of the Order of Leopold (Chevalier de l’Ordre de Léopold), given by Leopold I, the King of the Belgians in 1846; Knight of Our Order of the Oak Crown (Chevalier de Notre Ordre de la Couronne de Chêne) given by King William III of the Netherlands, Prince of Orange-Nassau and Grand duke of Luxembourg in 1849; Knight of the Order of the Danes (Ridder af Dannebrogordenen) given by his Majesty the Frederick VII King of Denmark in 1850; Knight of the Order of the Württemberg Crown (Orden der Württembergischen Krone) in 1852; Knight of the Military Order of Christ (Cavaleiro da Real Ordem Militar Portuguesa de Nosso Senhor Jesus Christo) given by King Pedro V, regent of the Kingdom of Portugal, in 1854, and; the great gold medal of the Russian Empire conferred by the Royal Imperial Majesty in 1857. Her Majesty the Queen of the Belgians, the King of Naples, and the Grand Duke of Tuscany sent him special testimonies of their royal appreciation.

## 6. Morren’s Teachings Spread Globally

Word of Morren’s success in pollinating *Vanilla* spread like wildfire across Europe. Between 1837 and 1839, his findings were published and transcribed in several agricultural and botanical magazines, stirring up horticulturists and scientists across the continent. The first to succeed in employing Morren’s method was Joseph Neumann, head gardener at the *Jardin du roi* (now *Muséum National d’Histoire naturelle*) in Paris. Neumann was without a doubt familiar with Morren’s work, given that he had been an active member of the Royal Horticulture Society of Paris when Morren’s discovery was presented as a novelty by Berlèse and Poiteau to the members of the Society [57,58].

Some authors have mistakenly suggested that Neumann, who first published his findings in 1838 not 1830, as erroneously cited by Delteil [22,61], claimed priority over the discovery of *Vanilla* pollination. But Neumann presented his work only as complementary observations and never claimed to be the first in succeeding to obtain *Vanilla* fruits artificially [62,63]. Despite not explicitly citing Morren, Neumann in fact referred to the Belgian’s prior findings. In 1839, Neumann wrote: “This plant, in the Botanical Garden of [Liège], produced long fruits, about 7 inches, the size of a child’s finger” and “The *V. planifolia*, which flowered last year in Belgium, has a flower very similar to that of the species which concerns us *” [63]. There was no priority dispute between the Belgian and French scientists over *Vanilla*. In fact, Morren was highly regarded by the members of the Royal Society of Horticulture of Paris, and in February of 1838, following his work on *Vanilla*, he was proposed and accepted as a corresponding member. We find more evidence that there was no dispute regarding priority in the journal of the Royal Society of Horticulture of Paris itself. In July 1838, Mr. Poiteau offered a summary of the situation. His text reads: “It is remembered that *Vanilla* fruited for the first time in Europe in 1836, in one of the greenhouses of the botanical garden of Liège. In 1838, a Vanilla… flowered and bore fruit in Paris, in one of the greenhouses of the Jardin des Plantes *” [64]. In July of 1840, Viscount Héricart de Thury wrote in the same journal: “The tests of Mr. Professor Morren, in Liège, of Mr. Neumann, in the garden of the natural history museum, can leave no doubt about the success of the cultivation of vanilla in our greenhouses *”, to which he added a personal note by Neumann himself stressing the importance of the time of fertilization [65].

Contrary to modern speculation, news of Morren’s success traveled far. His work was featured notoriously in papers by the Royal Academy of Science of Belgium, the Royal Horticultural Society of Paris, and the British Association for the Advancement of Science. He had a large network of correspondents in several countries, often including renowned figures of the time. Morren had been commissioned by the Belgian government to study the botanical gardens of Great Britain, and it was while visiting England, Ireland, and Scotland that, in 1838, he read the results of his studies on the pollination of *Vanilla* before the British Association for the Advancement of Science. His success in obtaining the precious aromatic fruits made such a splash that it motivated leading British botanist John Lindley to name the genus *Morrenia* Lindl., in the family Apocynaceae, in Morren’s honor that same year. The record of his reading before the association at Newcastle was published in the *Annals of Natural History* in 1839 [56]. By the early 1840s, more than a dozen publications written in English, French, German, and Italian referred to Morren’s incredible feat. In September of 1841, he personally attended the third meeting of Italian Scientists in Florence (Firenze), again showcasing his results. Shortly after Morren’s presentation in Florence, *Vanilla* pollination records surfaced in Italy, notably at the hand of Professor Roberto de Visiani. Visiani’s successful pollination of *V. planifolia* at the Botanical Garden of Padua has also, at times, been suggested to be an independent discovery of the artificial pollination of *Vanilla*. But this is not the case. Not only was Morren’s presentation published in the proceedings of the meeting in Florence [66], but Visiani clearly states that Morren’s and Neumann’s experiments are used as inspiration for his own work [53]. In a letter dated 26th June 1843, Visiani mentions that he received the gold medal from the Imperial and Royal Viennese Horticultural Society (*k. k. Wiener Gartenbaugesellschaft*) for being the first to successfully obtain Vanilla fruits in culture in the Austrian territory [53].

News of the successful hand pollination of *Vanilla planifolia* in Europe inevitably also reached the Dutch and French tropical colonies. The Dutch had introduced *Vanilla* into Java in 1820 and 1840, the latter most likely also included the knowledge of how to pollinate the plant. The second introduction of *V. planifolia* from The Netherlands to Java failed to produce a *Vanilla* culture, given the untimely death of Pierot shortly after arriving with the plants. However, the plants themselves were confirmed to be growing well and bloomed by 1844 [51]. Johannes Elias Teijsmann, head gardener of the Lands Plantentuin in Buitenzorg (now Bogor), pointed out that news had finally arrived on how to fertilize the plants, and this could be applied to expand its culture in Indonesia. In a letter by Simon Binnendijk dated 24th October 1850, the assistant gardener confirms they are “happy to see all the plants that bloom full of fruiting racemes”. Teijsmann and Binnendijk succeeded in implementing the pollination method and were the first to successfully set up a productive *Vanilla* culture in the Dutch colony of Java. This success prompted others in the region to imitate them, resulting eventually in an oversupplied *Vanilla* market [50]. Arditti [20,36] and Arditti et al. [67] point out that the Dutch did not disclose the method used in Indonesia, suggesting they could have developed one of their own. However, Antwerp, Ghent, and Liège had all been part of the United Kingdom of The Netherlands, separated after the Belgian Revolution in 1830, and not recognized as independent by The Netherlands until 1839. This was happening at the very same time as Morren’s experiments on *Vanilla*. During that period, agricultural and botanical material, knowledge, and technology continued to be frequently shared among Dutch and Belgian scientists and institutions.

It is the botanist Willem Hendrik de Vriese who confirms that the method followed by the Dutch in Java is that of Morren. He credits Morren as the discoverer of *Vanilla* pollination, pointing out that his breeding experiments in Liège are to thank for its culture in Europe and outside of it [49]. Gorkom [50] also notes that the work of Teijsmann and Binnendijk on *V. planifolia* in Java was based on the teachings of Morren. Suggestions that the Dutch in Java had independently discovered a method of pollination of *Vanilla* are, therefore, unfounded. However, they did find a way to apply this method successfully, so much so that thanks to their work, the *Vanilla* culture expanded strongly in Indonesia [49]. Regarding *Vanilla*, Teijsmann [68] wrote: “On the grounds that it must be artificially fertilized, men experimented and finally succeeded in discovering the method of fertilization, as a result of which all flowers now set fruit, so that they will soon be able to cultivate it on a large scale and Java will produce vanilla in abundance in the future”. The mention of a successful pollination attempt by Johannes van den Bosch, governor general of Java [20,36,67], should instead be ascribed to Van der Pant [69]. Van der Pant carried out his experiments on the estate of Governor Van den Bosch and was, in all likelihood, well aware of the previous achievements of Teijsmann, who had traveled to Java as the gardener to the governor.

Despite the famous claims of independent discovery in the French colonies of Réunion, Guadeloupe, and Martinique, this is not only unlikely but there is evidence to prove it untrue. It is important to remember at this point that both Morren’s and Neumann’s works of 1837, 1838, and 1839 had been mainly published in French journals and in the French language, featuring, among others, prominently in the journal of the Royal Horticultural Society of Paris. Given the close relationship between the colonies and Paris and the extensive and continuous exchange of people, goods, crops, technology, and information, it seems rather unlikely that important agricultural developments presented on tropical crops in the capital would somehow elude the tropical French colonies where those crops could become productive. We do not need to speculate about this as there are records of at least two introductions of the knowledge on *Vanilla* pollination from France into Réunion shortly after its discovery by Morren.

## 7. Perrottet, the *Comité d’Agriculture,* and Their Correspondents

At the beginning of the nineteenth century, the French—much like the Belgians, the British, and the Dutch—were in the habit of sending out emissaries to procure and develop promising agricultural products from their colonies. One such emissary working for the French government was Georges Guerrard-Samuel Perrottet, a French plant collector, experimental botanist, and biologist of Swiss origin. After working as a gardener, Perrottet became the naturalist of an expedition led by Captain Pierre Henri Philibert between 1819 and 1821. His duties were to collect rare and useful plants from Réunion, Java, and the Philippines to cultivate them in French Guiana. Between 1824 and 1829, he explored Senegal and Gambia, introducing the nopal cacti to raise dye-yielding scale insects. Between 1834 and 1837, he was appointed agricultural botanist at the Botanic Garden of Pondichéry in India, carrying out botanical collections and experimentation. On his expedition, returning from India to France, he collected plants in the Malabar Coast, the Nilgiris, Poona, and Bombay between 1837 and 1839. Back in Paris, Perrottet was commissioned by the Ministry of Marine and Colonies Department of France to activate the silk industry in the colonies of Bourbon [Réunion], Cayenne, Martinique, and Guadeloupe, spreading the mulberry trees and the methods to rear silkworm [70,71].

**Figure 5:**
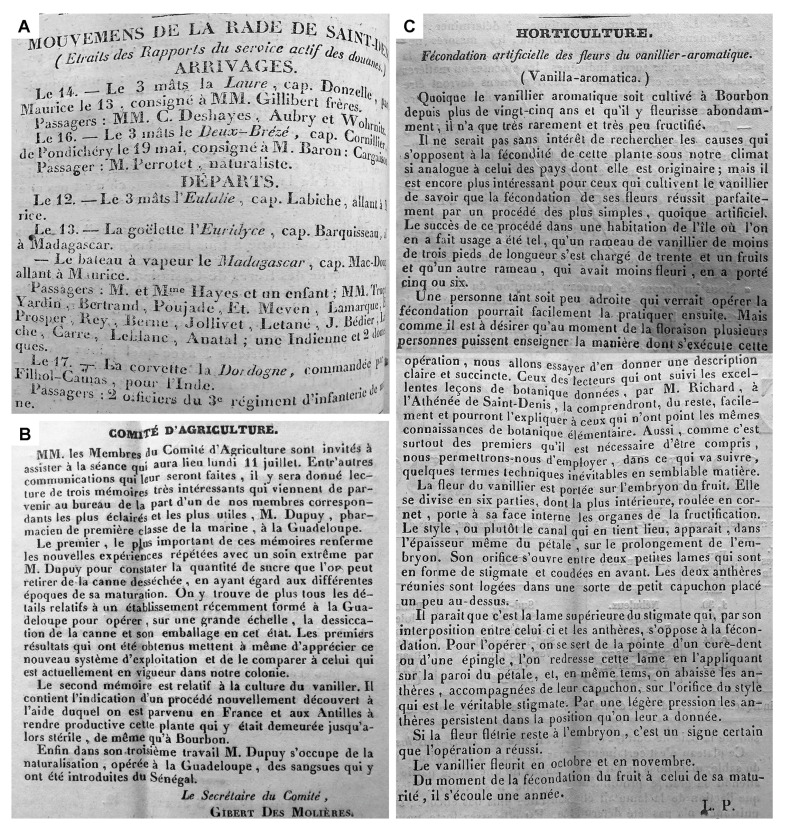
Reproductions from the *Feuille Hebdomadaire*. (**A**) Perrottet’s arrival recorded on Réunion island on the 16th of June 1839. (**B**) The *Comité d’Agriculture* announces that it has received a memoir by Mr. Dupuy relating to the culture of vanilla, it was read before its members on the 11th of July 1842. (**C**) Possibly the first publicly available description of Edmond’s pollination technique published on the 30th of August 1843. Photographs by the author.

Perrottet arrived once more on Réunion island on the 16th of June 1839 (Figure 5A), spending four months touring the island per request of the governor Anne Chrétien Louis de Hell. His duties included visiting farmers in the various districts across the island and giving advice to those inhabitants who would show the desire to cultivate mulberry trees and engage in the silk industry. Perrottet, signing as *Botanist agriculteur* to the government, presented a report on the silk industry on the island to the Minister of the Navy and Colonies and Governor De Hell. The report is offered by the governor to the members of his recently created *Comité d’Agriculture* of Réunion. The committee agrees to publish Perrottet’s memoirs, and he is appointed as a corresponding member. Perrottet returned to India and became the director of the Botanic Garden of Pondichéry from 1842 to his death in 1870. His influence in the development of agricultural products in the French colonies was instrumental. He is credited with introducing and developing numerous new crop species to France and its colonies, among his many introductions, we may cite the mulberry, *Morus multicaulis*, the Cayenne pineapple, *Ananas comosus*, and, of course, *Vanilla*. His experiments in India led to the establishment of commercial tea plantations, and besides the rearing of silkworms, he was also involved with dye-yielding plants and the extraction of the dye in the French colonies [70]. Furthermore, Perrottet was in the middle of a tightly knit web of communicating naturalists.

George Samuel Perrottet was a known correspondent of French botanist Achille Richard, who had also been a botany professor of Charles Morren during his studies in Paris in 1830 [40]. Achille was well connected to several of Morren’s acquaintances at the Muséum National d’Histoire Naturelle, including Delessert and de Jussieu, and was also a known correspondent of Méziéres Lépervanche [72], who was instrumental in Edmond Albius’ upbringing, as we will see below. Perrottet had a specific interest in orchids of the Indian Ocean, publishing monographic treatments for the Orchidaceae of Réunion [73] and, in collaboration with Perrottet, India [74]. A major corresponding network can also be established between George Samuel Perrottet, Jean Michel Claude Richard (unrelated to Achille Richard), Méziéres Lépervanche, and Charles Morren since all four men were active correspondents of French pharmacist and botanist Charles Gaudichaud-Beaupré. As director of the botanical garden on Réunion island, J.M.C. Richard knew both Lépervanche and Perrottet, the latter being a close friend [75]. J.M.C. Richard and Lépervanche were both active members of the *Comité d’Agriculture* on Réunion island, while Gaudichaud-Beaupré and Perrottet were both named corresponding members. During his voyage around the world on the *Bonite* (1836–1837), Gaudichaud-Beaupré visited Perrottet in Pondicherry, India, and visited J.M.C. Richard in the Mascarenes. Conversely, Charles Morren visited Gaudichaud-Beaupré in Paris in August of 1841 [40]. Another important link exists between Méziéres Lépervanche and Charles Morren via their mutual correspondent, French naturalist Charles-François Brisseau de Mirbel, whom we know the latter had personally informed of his *Vanilla* experiments [41].

These connections, albeit suggestive, do not offer definitive evidence that news regarding *Vanilla* pollination had indeed reached Réunion island from abroad. Hard proof came when a letter by Mr. Perrottet [76] written on the 30th of April was published in the *Moniteur officiel* of Pondichéry on the 4th of May 1860. It reads: “As for the fertilization of vanilla, I had spoken about it to several people on my last trip to the colony towards the end of 1839, in particular to MM. Pattu de Rosemont [sic], Beau-Mont [sic], l’Épervanche [sic], Méusière, distinguished botanist, etc., etc. I indicated the method followed by Mr. Neuman [sic], in the hothouses of the History Museum natural in Paris. These gentlemen may perhaps remember it. Besides, the method is simple and within everyone’s reach, it is enough to know how to distinguish the organs of generation, recognize the stamen, male organ, capped, and the labellum, female organ, which is welded to the gynostema or support of the anther; but it was necessary to put it into practice; and the one who first operated it on a large scale, must have the merit, if not of its discovery, at least of its practical use *”. In other words, not only does Perrottet claim it is he who personally brought news of Neumann’s artificial pollination of *Vanilla* to Réunion in 1839, but he specifically brought this information directly to Patu de Rosemond, Bellier-Beaumont, and Mézières Lépervanche.

The role of Perrottet in transmitting this knowledge to the planters on Réunion island has often been disregarded for reasons that are as yet unclear to me. The thorough works of Volsy Focard [77,78] fail to mention his statement on this issue, despite referring to this very same letter when discussing the matter of Perrottet’s influence on introducing *Vanilla* cultivation to Réunion. Delteil originally credits Perrottet as having transmitted the procedure of Neumann to Rosemond, Beaumont, and Lépervanche during his trip to Réunion in 1839 [61], but in a later publication, this information is omitted [22]. A few authors, such as Aimar [79], do credit Perrottet for having brought the information regarding Neumann’s experiments to the island. He certainly had the means, motive, and opportunity to do so. After being commissioned in France, Perrottet visited Réunion in 1839, a year after the publication of Neumann’s successful artificial pollination of *Vanilla* in Paris. His main duty was to introduce crops and spread agricultural technology in the French colonies. During his four-month stay in Réunion, we know that he extensively visited farms across the island. We also know he was well connected, being closely involved with the *Comité D’Agriculture*, which among its senior members included his close friend Jean Michel Claude Richard, and listed among its most active members Mézières Lépervanche. We know for a fact that Neumann [63] was aware of Perrottet’s hand in disseminating *Vanilla* plants, and there is no reason to doubt Perrottet would have become aware of Neumann’s work as well.

The second documented introduction of the discovery of *Vanilla* pollination into Réunion island occurs at the hand of Pierre-Sébastien Dupuy, a naval pharmacist in Guadeloupe [61,80]. In July of 1842, the *Comité D’Agriculture* announced that it had received three interesting memoirs by Mr. Dupuy, one of its corresponding members. In the meeting on the 11th of July 1842, Mr. Dupuy’s memoir relating to the culture of vanilla was read before the members of the *Comité D’Agriculture* (Figure 5B). It contained critical information on “a newly discovered process, with the help of which it was possible in France and the Antilles to sell productively this plant which had until then remained sterile, just as in Bourbon”. It is uncertain if Mr. Dupuy, prior to offering these memoirs, visited and informed farmers on Réunion island of this technique (as had been done by Perrottet), but it is certainly possible. A Mr. Dupuy, listed as navy sublieutenant, is recorded to have arrived in Réunion on the 17th of April 1841, under Captain Ferrin, and was returning to the port of Toulon on the *Iphigénie* after visiting Rio de Janeiro. Mr. P. Dupuy is also recorded to depart from Réunion in March of 1842. Perhaps neither of these is Pierre-Sébastien Dupuy, but we know for certain that his manuscript made it to the island.

Dupuy did not discover another method of *Vanilla* pollination. He served as a messenger for a pollination technique that had been successful elsewhere in France and the Antilles. There is evidence that the knowledge regarding *Vanilla* pollination had reached the French Antilles by 1839 where it had been brought by Daniel Beauperthuy, a Creole born in Guadeloupe. Beauperthuy had an interest in natural sciences, he attended courses in botany and entomology and graduated from his medical studies in Paris in 1837, being appointed traveling naturalist (naturaliste voyageur) to the Muséum National d’Histoire Naturelle [81,82]—this is the very place and time when Neumann carried out his experiments on *Vanilla* pollination [62,63]. After his stay in Paris, Beauperthuy returned briefly to Guadeloupe in 1839 [83] before settling finally in Venezuela. He is credited with having introduced the knowledge of *Vanilla* pollination into Martinique, surely contributing to the several hundred kilograms of *Vanilla* being exported from the colony as of 1840 [84].

As we will see below, Edmond Albius is often credited as the “true and only” discoverer of the artificial pollination of *Vanilla*. It is suggested that Edmond—an enslaved teen at the time—was the victim of unscrupulous botanists trying to rob him of the credit for the groundbreaking innovation. Given the unjust, defenseless circumstance of Edmond, it is not surprising that most would jump to his side when the issue of who can claim priority over *Vanilla* pollination arises. But this dispute originates in what I regard as a series of misinterpretations. One may wonder why Edmond Albius is so often regarded as the discoverer of artificial pollination in *Vanilla* when we know with certainty that the efforts of Morren, Neumann, Beauperthuy, Perrottet, Pierot, Dupuy, and Visiani, predate his technique becoming public.

## 8. Edmond Albius, Mézières Lépervanche, and Bellier-Beaumont

Edmond was born in 1829. His mother, who died during childbirth, was a slave to Madame de Bellier-Beaumont. Edmond was transferred to her brother, Féréol Bellier-Beaumont, and eventually became “his favorite”. The Bellier-Beaumont family were educated and well-connected plantation owners in Sainte-Suzanne, on the east coast of Réunion island. Edmond had no school education but had a personal interest in botany and horticulture. He was a so-called ‘specialized’ enslaved group [85] working as a gardener for Bellier-Beaumont. Edmond often studied and experimented on cultivated plants together with Mézières Lépervanche, a nephew of Féréol, who was raised by his uncle after he lost his father during childhood. Bellier-Beaumont and, especially, Lépervanche, initiated Edmond in botanical studies, and they would have instructed him on experimenting with fertilizing Vanilla flowers [72]. Lépervanche devoted himself to the study of natural history and became a trusted correspondent of many members of the French Academy of Sciences. His involvement is critical, not only for being the formally trained and well-connected botanist of the group but also for being vocal on the role of Edmond in finding a practical way to pollinate *Vanilla*.

The first printed record of Edmond Albius’ name in relation to the pollination of *Vanilla* is in the article *Introduction et Fécondation du Vanillier à L’Ile Bourbon* by Volsy Focard [77] published on the 10th of October 1862. It is here, that based on an exchange of letters between the author and Patu de Rosemond, Bellier-Beaumont, and Mézières Lépervanche, that Focard puts forward the claim that *Vanilla* pollination was discovered by Edmond Albius in 1841. The earliest written record of Edmond’s name being linked to the discovery of the procedure to pollinate *Vanilla* in Réunion is a letter dated 8 December 1853, addressed by Mézières Lépervanche to the governor. In this letter, which has been reproduced in part by Focard [77] and fully by Chabin [86], Lépervanche mentions that Edmond was sent to the estates of Mr. Patu de Rosemond, Mr. Floris, Mr. Desbassayns, and Mr. Vinet to spread the knowledge on vanilla fertilization first hand.

But what does Lépervanche actually say in the letter? One crucial element of the 1853 letter is that he does not claim that the “fertilization of *Vanilla* was unknown in the world before Edmond *”, in fact, Lépervanche clearly states that “it had already been practiced ten years previously in an orangery in Brussels [sic], and at the Natural History Museum in Paris *”. This points to the fact that the works of Morren and Neumann were previously known in Réunion, as expected by the network of corresponding naturalists, and confirmed in Perrottet’s statement and Dupuy’s memoir. But, Lépervanche clarifies, “this discovery had only had an echo in the scholarly world, and the process used was much less simple *”. Consistently, a letter by Bellier-Beaumont dated 9 December 1862 reads: “In 1837, we had already managed to make the *Vanilla planifolia* bear fruit by a process different from that of Edmond. We cut the labellum that forms an operculum with the stigmatic surface. Mr. Richard, at the Paris Plant Garden, must naturally have been informed of the discovery made in Liège *” and “before Edmond, no one, even Mr. Richard, had practiced fertilization in Bourbon *”. From this, we must conclude that the original claim by Lépervanche and Bellier-Beaumont was not that Edmond had discovered *Vanilla* pollination, but rather that he had found and disseminated a practical technique to implement it in Réunion.

The second mistake introduced by historians is the 1841 date. In the letters by Bellier-Beaumont and Lépervanche, both state that they are unsure about exactly when the events transpired. This makes sense, Lépervanche’s letter came a decade after the fact, while Bellier-Beaumont was discussing events that had transpired some 20 years before. On the 17th February 1861, Bellier writes “Although I have before me the draft of the note that, in time I sent to the editor of the country’s first newspaper, Mr. de Montmarqué, having neglected to date it, I cannot tell you very precisely the year of the discovery. But if you want to know it exactly, consult the diary written by Mr. de Montmerqué from 1840 to 1842. My memories and those of the young black man, author of the discovery, point us to 1841”. Historians have simplified this into making the 1841 date a fact. We must consider that the arrival on Réunion island of the recipient of this letter, Mr. Montmerqué, is recorded by the local newspapers to have occurred on 12 September 1842 (as Montmerquier), and he did not start being involved as an editor for the local paper *Feuille Hebdomadaire* until early 1843. Therefore, Bellier-Beaumont’s letter to the editor was sent at the end of 1842 or, more likely, on or after 1843. The discrepancy in dates may explain why no one has been able to locate this letter in the local newspapers of the time. I personally searched in issues of the *Annales du Comité D’Agriculture*, *Feuille Hebdomadaire* and *L’indicateur colonial de l’île Bourbon*, (the latter two afterward were fused, becoming *Le Moniteur*) from the years 1839 to 1843 and found no references to Edmond, Bellier-Beaumont or Lépervanche in association with *Vanilla*.

The latter is especially relevant because Lépervanche, being a corresponding member of the *Comité D’Agriculture*, actively wrote on agricultural crops in the *Annales du Comité D’Agriculture* during that time [87,88]. That he would fail to mention Edmond’s breakthrough regarding vanilla is inexplicable, unless this happened afterward. In fact, notes and reports published by the *Comité d’Agriculture* between 1839 and 1843 in both the *Feuille Hebdomadaire* and the *Annales du Comité d’Agriculture* support the hypothesis that, even if Edmond pollinated *Vanilla* at the Beaumont farm somewhere between 1840 and 1842, this did not become of public knowledge until later (it is important to mention that *V. planifolia* fruits can take from nine months to a year to mature). A letter dated 1st of November 1841, published in the *Feuille Hebdomadaire* and signed with the initials “L.B.”, points out that transforming *Vanilla* from an ornamental to a productive plant could represent a source of incalculable wealth for Réunion and asks the Agriculture Committee to highlight the advantages of its culture given that the plant has shown “rapid and easy reproduction” on the island, and “its fruits too *” [89]. The letter is important because it allows us to assess what was common knowledge in Réunion at the time. It suggests that the successful pollination of *Vanilla* was indeed already known but makes no special mention of any particular developments in this regard at the Bellier-Beaumont estate. It is shortly after this explicit written request that the *Comité d’Agriculture* announced having received Mr. Dupuy’s memoir regarding *Vanilla* culture, which points to the committee itself actively searching for assistance in implementing its production on the island. It is not until the 30th August 1843, in the article *Fécondation artificielle des fleurs de vanillier-aromatique* that appeared in the *Feuille Hebdomadaire* (Figure 5C), that we find what is possibly the earliest evidence of Edmond’s practical method of pollinating *Vanilla*. The text is signed with the initials L.P., and it reads, “the fertilization of its flowers succeeds perfectly by a very simple, although artificial, process. The success of this process in a house on the island where it was used was such that a vanilla branch… was loaded with thirty-one fruits *”. It carefully explains the procedure, indicating that with the “point of a toothpick or a pin *” the rostellar flap is straightened, allowing the application of the pollen on the stigma using “slight pressure” [90]. As far as I can tell, this is the first time the Edmond technique was made public.

Edmond has famously been said to have toured *Vanilla* plantations across Réunion, teaching his technique. I would argue that this did not happen before the second half of 1843 rather than in 1841, as suggested by most historians [77,80,85,91]. The later date, 1843, is in line with (1) the written request to the *Comité d’Agriculture* in November of 1841 to explore *Vanilla* production as an agricultural novelty; (2) Dupuy’s memoir presented to the *Comité d’Agriculture* in July 1842; (3) Bellier-Beaumont’s letters, suggesting Edmond showed him the practical technique sometime between 1840 and 1842, meaning the first experiments could have occurred in the flowering season end of 1842; (4) the fact that the letter of Bellier-Beaumont was addressed to Montmerqué, and therefore could not have been published before 1843 when he began his work as editor; and (5) that *Vanilla* exports from Réunion island are being recorded for the first time in 1846, amounting to 38 kg, according to records at the *Archives départementales*.

The final historical misconception is the myth that Morren’s method was complicated, impractical, and restricted to scholars. A few examples are provided. “[The Morren method consists of] cutting the labellum which forms a mobile operculum on the stigmatic surface, and prevents its powdery pollen mass to fertilize the female organ”. “He [Albius] did more than simplify the difficult procedure of Mr. Morren *” [92]. “The Morren technique consists of removing the pollinia with a needle and inserting them between the rostellum and the stigma” [93]. “The problem with Morren’s method of fertilization was that he used a pair of scissors to cut the rostellum, a relatively fiddly technique suited only for greenhouse conditions. His discovery, while academically important, remained a subject for discussion only within the narrow circle of academic botanists *” [92]. We learned previously that there is compelling evidence showing Morren was well connected and had been quite vocal about and interested in the implications of his discovery well outside academic circles. But what about the idea of his method being impractical? When consulting Morren’s original description of the process, we quickly learn he never circumscribed the method in the manner alleged. Morren [55] stated: “**Upon lifting or removing the apron** and the entire pollen mass, or just a part, is brought into contact with the stigma… many flowers can be fertilized *” [bold added] and later, again Morren [56] explains: “**It is thus necessary either to raise the velamen or to cut it** when the plant is to be fecundated, and to place in direct contact the pollen and the stigmatic surface…” [bold added]. Contrary to the common misconception that methods used elsewhere were “highly complex and with a poor record of success” [94], the records tell another story. The number of fruits initially reported at the Bellier-Beaumont estate [90] is lower than those obtained earlier in Liège [56,58], and while Martinique was exporting over 300 kg of *Vanilla* in 1840 [84], Réunion would not reach such a number until the 1850s.

The persistent idea of the inefficacy of Morren’s method surely originates from the writings of Volsy Focard, who echoed the words of Lépervanche [77]. Lépervanche probably actually believed other methods were impractical because the information on how to pollinate *Vanilla* arrived on Réunion indirectly via Perrottet and Dupuy. It is unlikely that the original texts of Morren made it to the island at the time. Neumann [63] mentioned that the fertilization of *Vanilla* flowers was not easy and required small forceps, and it is straightforward that Lépervanche would therefore assume the previous method was impractical, especially if he was not familiar with Morren’s work. This unawareness implies that Edmond did indeed figure out a way to take the teachings of the French naturalists—based on Neumann—and develop an effective and practical method of pollinating *Vanilla* flowers that could revolutionize its culture on the island.

## 9. Edmond’s Revolution

Edmond’s contribution was far from minor. He may not have discovered how to pollinate *Vanilla*, but his mastering and spreading of a practical method to fertilize the flowers was paramount for the development of a productive vanilla culture across the Réunion and the French colonies. Dispensed from the difficulties in growing *Vanilla* in the temperate climate of Europe, and the difficulties in getting it to flower regularly, the French tropical colonies provided the perfect setting for a quick expansion. Réunion went from exporting 0 kg of vanilla in 1841 to 36 kg in 1846 to 267 kg in 1853, and over 3 tons in 1858, and soon would become the world’s leading exporter [94].

Located in the Indian Ocean, over seven hundred kilometers from Madagascar, Réunion was a haven for those traveling the route between Europe and India. It had a tradition of plant acclimatization by naturalists and administrators looking to diversify the crops for economic development and was an important slave trading route. During the colonial period, slavery contributed significantly to the agricultural expansion, and the start of vanilla culture on the island coincided with the abolition of slavery. Emancipation occurred in 1848, and it had a great impact on all branches of agriculture, including *Vanilla*, and was often met with resistance from plantation owners [94]. We know from a letter conserved at the *Archives* that by 1849, vanilla growers such as the Desbassayns were seeing their farms and livelihoods threatened. Madame Desbassayns lamented the lack of workers after the emancipation and the “inability to afford the wages of the agricultural workers”, for which she requested financial aid from the General Commissioner. Edmond, too, became a free man, taking the surname Albius and leaving the Bellier-Beaumont estate and his vanilla days behind.

The name Edmond Albius still carries an important weight in the cultural and agricultural history of Réunion [85,86,94,95]. Edmond’s role in finding and, especially, teaching a practical technique for artificially fertilizing *Vanilla* flowers is undisputed. It is thanks to him that the commercial exploitation of *Vanilla* overtook Réunion at a time when the island was in need of new agricultural products. That a slave with little to no formal education could make such a big impact on the livelihood of so many people certainly has enormous merit. Given his humble background, he has become an inspiration for young people, and rightly so. It is no surprise then that an important technological advancement implemented by a slave would serve to reinforce human inventiveness, as well as social justice. *Vanilla* today is a symbol of cultural pride on the island, and it is no wonder that a picture or drawing of Edmond hangs on the wall everywhere that it is being cultivated, processed, or sold.

**Figure 6:**
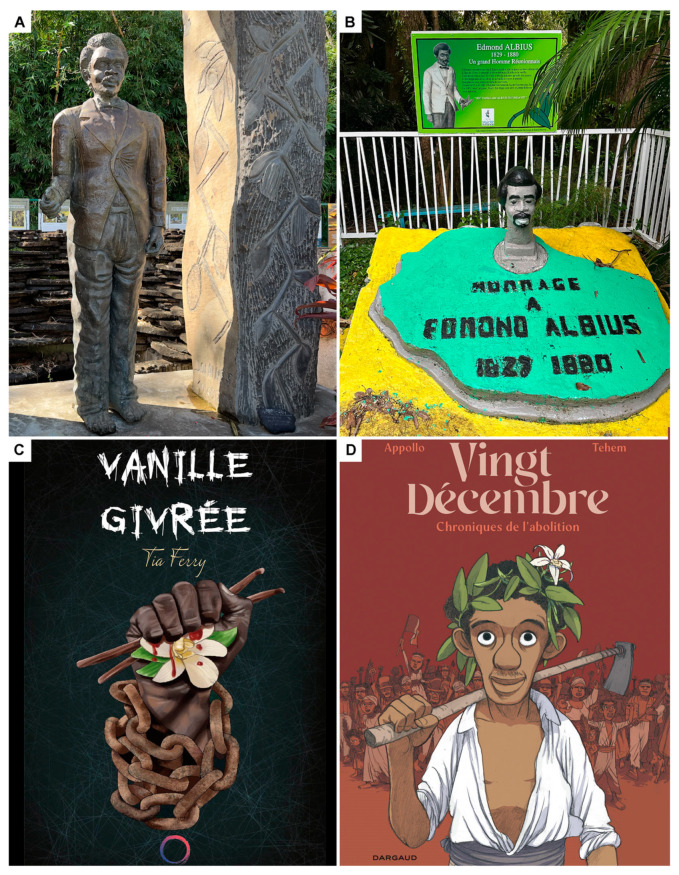
Edmond Albius, an important socio-cultural icon on Réunion. (**A**) His memorial includes a large bronze sculpture of the slave with *Vanilla*. (**B**) Homage at the entrance of the original Bellier-Beaumont farm in Bellevue, Sainte Suzanne. (**C**) The cover of *Vanille Givrée*, a thriller by Tia Ferry, features a bloody, chained fist holding two vanilla fruits and a flower. (**D**) *Vingt Décembre, chroniques de l’abolition* tells the story of the abolition of slavery through a brilliant young slave who discovers the process of fertilizing vanilla but is exploited by his rich owner. Photographs by the author (**A**,**B**), ©Ozril Éditions (**C**), ©Dargaud (**D**).

Réunion effectively transformed the exotic spice into a lucrative commodity, pioneering the technological breakthroughs required for mass production [94,95], and this began with Edmond’s revolution. On 10th May 2004, a memorial to honor Edmond Albius was installed at Le Bocage in Sainte Suzanne, Réunion. The memorial includes a large bronze sculpture of the slave holding a Vanilla (Figure 6A) placed in the middle of a garden of Vanilla plants and panels with information on the discovery. It also presents diverse social manifestations regarding slavery and social justice. An homage is also found at the entrance of the original Bellier-Beaumont farm in Bellevue, Sainte Suzanne (Figure 6B). Edmond has become an important socio-cultural icon, a symbol of the fight for emancipation. The story of an enslaved Creole teen making such a crucial contribution to the agricultural development of Réunion at the time of the abolition of slavery and how this would later grant him his freedom after being in prison has inspired both thrillers (Figure 6C) and chronicles on abolition in the form of a social comic (Figure 6D). It is no surprise that each town on Réunion has an Edmond Albius road, and a few schools on the island carry his name.

## 10. Setting the Bases for Turning *Vanilla* into a Global Commodity

It is often repeated that a single person has been responsible for turning the *Vanilla* vine from a sterile plant into a global commodity worth 1.26 billion dollars today [1]. In reality, the history of how *Vanilla* came to conquer the world is a story in which an extensive network of many hands played parts, each step becoming the foundation for the next (Table 1). From the gardeners who patiently cultivated the plants, to the ship captains who carried them in their boats, from the administrators who made conscious decisions with the nation and colonies in mind, to the naturalists that executed these decisively for the good of the people, from artists, and farmers to explorers, the curious, the stubborn and the motivated, countless people shaped the history of *V. planifolia*. Those who worked decisively and persistently in laying the foundations for *Vanilla* culture into what it is today are listed hereafter.

*Charles François Antoine Morren (1807–1858)*. Belgian naturalist, known especially for his work as anatomist, botanist, and horticulturist while occupying the botany chair and being director of the Jardin Botanique de l’Université de Liège. A prolific experimental botanist, in 1836 Morren became the first person on record to artificially pollinate *Vanilla*. His method soon became known globally, revolutionizing *Vanilla* production. Techniques that put the method into practice were spread by different people in the tropical colonies of The Netherlands and Franc and became the precursors to the world’s current commercial production. The grave of Charles and Edouard Morren is located at the Robermont Cemetery in Liège, Belgium. A memorial, with a bust and original elements of his work on *Vanilla*, can be found at the herbarium of the University of Liège. A display case of his work can be found at the old Botanical Garden in the city center.

*Edmond Albius (*ca. *1829–1880)*. Creole slave from Réunion island. As a teenager, he figured out a practical technique for hand-pollinating Vanilla flowers, which he taught to farmers across the island. Thanks to him, commercial exploitation of *Vanilla* overtook Réunion at a time that the island was in need of new agricultural products. A monument dedicated to his influential development of *Vanilla* culture in Réunion has been placed on Pierre Mendes Avenue in Sainte Suzanne, Réunion. Another, more informal, memorial is found at the entrance of the Bellier-Beaumont farm in Bellevue, Sainte Suzanne. Edmond has become an important socio-cultural icon, a symbol of the fight for emancipation.

*Georges Guerrard–Samuel Perrottet (1793–1870)*. A Swiss-French naturalist employed as an agricultural botanist for the French government, collecting close to 10,000 plant specimens. Perrottet was instrumental in the introduction and development of new and interesting agricultural crops in the colonies, including Réunion, where he introduced *Vanilla* from Cayenne in 1819. In 1839, he visited the island again, spending four months touring its farms as per the request of the governor, giving advice to those who showed the desire to cultivate mulberry trees and engage in the silk industry. Among others, during that time, he also provided growers the necessary information to attempt artificial pollination of *Vanilla*. The genus *Perrottetia* Kunth is named in his honor. Perrottet’s grave and memorial can be found at the *Jardin Botanique de Pondichéry*, Puducherry, India.

*Johannes Elias Teijsmann (1808–1882)*. A Dutch botanist and gardener. In 1830, Teijsmann traveled to Java (Indonesia) as gardener of Governor General Johannes van den Bosch. He was then appointed head gardener of the *Lands Plantentuin Buitenzorg* (now Bogor). Teijsmann is credited with introducing numerous plants to and from the Dutch colonies in the East Indies, including cassava and cinchona. He set up the *Vanilla* culture in Java together with Binnendijk after pointing out that news had finally arrived on how to fertilize the plants and that this could be applied to expand its culture in Indonesia. A monument in his honor has been placed at Bogor Botanical Garden, Bogor, West Java, Indonesia.

## 11. *Vanilla* in a Changing World

A delicate balance exists between partner organisms that are involved in mutualistic relationships. Given the highly specific nature of these intricate relationships, biologists and ecologists have been concerned about the possible disruptive effects that habitat disturbance and climate change may have on the ecological interactions between orchids and their mycorrhizal fungi, host trees, pollinators, and seed-dispersers, among many other partners [33]. It is now well known that the continued transformation of our planet upsets the balance between partner organisms and threatens to provoke the uncoupling of important species interactions. The effects that climate change is having on flowers and their pollinators can already be measured, and one way to do so is through its potential to disrupt the synchrony of highly specific interactions between plants and insects. Specialist plants with few pollinator species are highly sensitive to this decoupling. Potentially, upsetting the delicate balance between partner organisms can destabilize entire ecosystems and can be a serious threat to human food security and ecosystem services in general. Such disruptions of pollination are already apparent among certain orchids [96] and are expected in others [97].

Besides pollen availability and presentation, another issue of equal concern is pollen viability, which is strongly affected by environmental conditions [98]. Although it remains unclear to what extent pollen will have the capacity to deal with environmental challenges, it is certain that climate change will have an impact on pollen production and pollination capacity [99]. The more packaged pollen is, the more it is protected from environmental damage [98]. Most orchid pollen is found tightly packed into single units called pollinaria which are composed of pollinia, and those in turn of pollen tetrads in differing conformations [100,101]. Unfortunately, this is not the case for *Vanilla* flowers, in which pollen is mostly found as granular, loose tetrads, rather than forming defined pollinia. Pollen degradation is a major concern for other crops, even those that do not require animal vectors for pollination.

Given that the *Vanilla* plant is vulnerable to dry spells, extreme temperatures, and excessive winds, such as cyclones, hurricanes, and tropical storms, the crop is highly sensitive to the impacts of climate change in most current areas of production [3]. At the turn of the century, prices quadrupled overnight on account of a single category-five cyclone devastating the vanilla-growing region of Madagascar [102]. By 2004, vanilla demand was half that of a few years before, resulting in a crash in prices that again affected the farmers [102]. It is expected that the vanilla crop will become even more vulnerable, leading to overall global yield declines [103,104]. Direct anthropogenic pressures, from agricultural intensification and land use change, also put a severe strain on the *Vanilla* crop’s wild relatives (CWRs), with most species already being endangered [103,105]. The reduction or overall unavailability of natural pollinators can have devastating consequences for the natural populations of wild plants in general. But it is also critical for many crops.

The dependence on artificial pollination of the vanilla crop is costly. In Madagascar, more or less 19% of the cost of vanilla production per hectare derives solely from pollination activity [106], while in Costa Rica, costs associated with artificial pollination can represent up to 32.25% of the yearly production costs [107]. Rising temperatures not only disrupt the natural pollination process. It may also render pollen altogether unviable and may have serious consequences on yield and fruit processing. In the case of *Vanilla*, high temperatures are known to cause the early abortion of fruits. On Réunion island, farmers are already worried about the effects of rising temperatures on crop production and the delicate curing process. At La Vanilleraie, a major player in the Sainte Suzanne area, *Vanilla* plants grown under greenhouse conditions are sprayed with water to lower temperatures and prevent fruit abortion [108]. Farmers like him ask that the government intervene by offering new land to growers to relocate their farms to higher elevations where the temperatures are cooler. This might be a short-term solution for these farmers, but it certainly cannot be sustainable in the long term.

A more sustainable approach to vanilla production was suggested by Watteyn et al. [109] in which a joint land-sparing and land-sharing approach was put forward for Middle America. This entailed protecting natural *Vanilla* populations and their pollinators inside forests while using some of the neighboring lands for vanilla cultivation in agroforestry systems where the natural interactions are kept as intact as possible. Understanding the genetic basis and diversity of *Vanilla* has also been identified as key for breeding programs. Among the key traits to look for in breeding programs, not surprisingly, we find self-pollination and higher yields, but also adaptation to hotter and drier growing conditions [3,110,111]. To these, we may soon need to add pollen durability as a desired feature in breeding programs involving *Vanilla*, as well as other crops. Immediate actions are required to conserve the genetic diversity found in the *Vanilla* crop wild relatives (CWRs) and, at the same time, attenuate the effect of extreme climates in vanilla culture.

Bramel and Frey [3] identified three strategic areas on which a global strategy for the conservation and use of *Vanilla* genetic resources should focus: (1) securing the long-term conservation of vanilla genetic resources; (2) increasing the availability and exchange of germ-plasm; and (3) increasing the use of the genetic diversity conserved. The author stressed the importance of creating opportunities for global collaboration building upon institutional and individual expertise in order to ensure these genetic resources. Taking collaborative action in these strategic areas is indeed badly needed, and important challenges remain due to significant gaps in our knowledge of the basic biological and ecological aspects of vanilla.

Many *Vanilla* species are considered rare or threatened in nature, having reduced population sizes. Prior efforts to look for genetic variation in search of promising traits have focused on Vanilla specimens, which are readily available in culture. These studies have identified relatively low genetic diversity among cultivated plants [112,113]. This is due mainly to the clonal reproduction of cultivated individuals, which stimulates rapid growth and sustained quality but leads to significant genetic erosion. Conversely, genetic variation in ex-situ collections and wild accessions of *V. planifolia* and its CWRs has been shown to be high [114,115]. Efforts should, therefore, aim to both locate and circumscribe this natural wild diversity in order to conserve and use it in a sustainable and socially ethical way. Uncertainty remains as to the correct identification of many vanilla specimens, both in herbaria and in culture [5]. This confusion with regard to identity is also widespread among cultivated materials [116], offering both challenges and opportunities for breeding programs. Ex situ germplasm collections, the basis for breeding, are mainly found outside the natural distribution of the crop and its wild relatives. I agree with Soto Arenas [117], Roux-Cuvelier and Grisoni [118], and Flanagan and Mosquera-Espinosa [105] that a global strategy for the conservation of local biodiversity, focusing on the centers of origin, is paramount. Prompting in situ and ex situ conservation in collaboration with the Middle American countries where *V. planifolia* populations are found naturally. This needs to be informed by research, especially regarding distribution and gene flow, genetic diversity, ecological preferences, and desirable traits. Only in this way can *Vanilla* breeding take a leap into the future, truly assessing and ethically and sustainably using the available natural variation that is found locally and answer to our current needs globally.

Despite representing a global market of over a billion dollars, the vanilla industry is highly dependent on impoverished farmers in developing countries [102]. Funds for conservation and research on vanilla in those countries also remain scant. For many institutions and activities targeting *Vanilla* conservation, funding was deemed inadequate and declining [3]. Latin American institutions, researchers, and students are rarely the recipients of any international grants to fund their research on vanilla diversity, ecology, and resilience despite their global necessity and potential impacts. Thanks to ongoing studies in Middle America, today we know important details as to how *Vanilla* species of commercial interest naturally pollinate [8,13,27], disperse, and germinate [24,25,119]; what host plants and fungal partners they maintain [120]; how genetically diverse wild *Vanilla* populations are [121]; and how climate change might affect both the plant [104,109] and its pollinators [122]. But much more needs to, and can, be done. Only by supporting and collaborating with these initiatives will we be able to offer the knowledge that is required to inform policies and strategies aimed at an efficient, sustainable, and ethical vanilla culture.

I would like to conclude this review by echoing the words of De Vriese [49], who, on speaking about the discovery of artificial pollination in *Vanilla*, pointed out that: “Men would not have been able to do those experiments, nor gotten those results, if not informed by science”. It is more true than ever that we can only build upon what science informs us. Speaking about the work of Morren, De Vriese continues: “All intertropical colonies will thus be able to produce vanilla. It will succeed in all greenhouses. It will serve as proof of the importance of science for industry everywhere. From now on, the cultivation of the *Vanilla* was assured of a good success in all tropical regions and one can therefore expect good success from the importation of that plant into all colonies”. His words proved to be prophetic, but the threats to wild *Vanilla* CWRs and likewise *Vanilla* culture are now greater than ever, and we need to look at science and especially collaborative research to find the solutions for *Vanilla* culture in a changing world.

Note: an asterisk * follows texts as interpreted by the author on the basis of the Google Translate software from the French original.

## Figures and Tables

**Table 1:** Historical events leading up to the discovery and implementation of artificial pollination in *Vanilla*.

Date	Historical Event
1793	Sprengel asserts that pollen masses need to be directly applied to the viscid surface of a stigma for flowers to become fertilized, and points out insects are the main agents behind this process [34].
1798	Wachter successfully pollinates an orchid artificially for the first time [35].
1800	*Vanilla planifolia* is introduced by George Spencer Churchill, the Marquis de Blandford [18]. It is later grown at the estate of Right Hon. Charles Greville in Paddington [42,43].
1804	Salisbury asserts he has been successful in artificially fertilizing several orchid species by applying the pollen masses directly to the stigma [37].
1807	*Vanilla planifolia* flowers for the first time in Europe at the estate of Right Hon. Charles Greville [42]. Illustrations are prepared by Franz Bauer [44], Sir William J. Hooker [43], and Andrews [45].
1812	*Vanilla planifolia* from Mr. Greville is introduced into cultivation by Joseph Parmentier, mayor of Enghien, and entrusted to the care of Claude Louis Sommé in the botanical garden of Antwerp, from where it was sent across Belgium and France [42,47].
1814	Dr. Sommé confirms that the *V. planifolia* from English origin grows well in the greenhouses in Antwerp, but that they have not flowered anywhere in Belgium except the at Ms. Viscount Vilain XIIII’s collection in Wetteren [47].
16 July 1818	*Vanilla planifolia* blooms at botanical garden in Ghent.
1819	Sommé provides Joseph Marchal, former employee in the Dutch East Indies from Brussels, with 5 potted *V. planifolia* cuttings to take from Antwerp to Batavia (Java) for acclimatization in the Dutch Indies [42,47].
1822	Marchant takes cuttings provided by L.A.G. Bosc from Paris to be cultivated at the estate of his mother in law, Madame Fréon at la Belle-Eau, La Réunion [52].
1829	Richard Courtois reports *V. planifolia* flowering at Liège and prepares a specimen [52].
November 1831	Robert Brown reads his work on the fecundation of orchid flowers before the Linnean Society of London [38].
1831	Adolphe Brongniart publishes Observations sur le mode de fécondation des Orchidées et des Cistineés [39].
1833	It is confirmed that *V. planifolia* blooms yearly at Padua Botanical Garden in Italy [53].
1834	Courtois reports *V. planifolia* climbing naturally on a fig tree and flowering every year at Liège.
July 1834	Naturalist Georges Guerrard-Samuel Perrottet visits Jean Michel Claude Richard, director of the botanical garden on Réunion island [75].
December 1834	The detailed sketches of *V. planifolia* by Bauer are published [44].
December 1835	Charles Morren occupies de botany chair at Liège after the passings of Gaëde and Courtois and becomes director of the botanical garden [40].
16 February 1836	The first *V. planifolia* flower is hand pollinated by Morren at Liège [41,55,56].
16 February 1837	The first artificially obtained *V. planifolia* fruit fully matures and naturally drops at Liège [41,55,56].
5 April 1837	Vice-president Lorenzo Berlèse and rapporteur Pierre Antoine Poiteau of the Royal Horticulture Society of Paris report on having observed the results of Morren’s work in Ghent and encourage him to publish his results.
May 1837	Morren’s article with news of the first artificially obtained fruits of *V. planifolia* at Liège is published after being read by Berlèse before the members of the Royal Horticulture Society of Paris [55].
September 1837	Camuzet visits Belgium and observes the fruits of *V. planifolia* at Liège and Leuven [59,60].
1838–1839	Neumann, an active member of the Royal Horticulture Society of Paris, publishes his own experiments on the pollination of *V. planifolia* in Paris, based on the teachings of Morren [62,63].
February 1838	Morren is proposed and recognized as corresponding member of the Royal Horticulture Society of Paris.
July 1838	Poiteau reports on *Vanilla* being hand-pollinated for the first time in Europe in Liège in 1836 and Paris in 1838 [64].
3 June 1838	Based on a report by Camuzet of 2.5 kg of *Vanilla* fruit produced recently, Soulange Bodin notes that what accomplished at the University of Liège could one day found a very lucrative branch of horticultural industry [60].
16 June 1839	Naturalist George Samuel Perrottet arrives on La Réunion from Pondichery and stays for 4 months.
2 September 1839	Perrottet, as Botanist agriculteur de Gouvernement, offers clarifications on a report he presented on the mulberry trees and the methods to rear silkworm on Réunion.
27 September 1839	Méziéres Lépervanche is appointed corresponding member of the Comité d’Agriculture of La Réunion.
15 October 1839	The section of new industries of the Comité d’Agriculture offers a report on Perrottet’s work on the industrie sérifére. The committee agrees to publish a memoir of Perrottet’s work.
July 1840	Viscount Héricart de Thury, president of the Societe Royal D’Horticulture de Paris and the Société royale et central d’agriculture, refers to the success of Morren and Neumann in cultivating and fertilizing *Vanilla* [65].
1 October 1840	The secretary of the Comité d’Agriculture of La Réunion reads a letter from Mr. Lépervanche on the culture of coffee, who has now concluded his studies on botany and other branches of natural history [87,88].
1 December 1840	The Comité d’Agriculture of La Réunion confirms having received samples of the memoirs of Mr. Perrottet, a corresponding member, on the culture of mulberry and soy.
1840–1841	Upon the recommendation by Blume and Reinwardt, the Dutch government purchases cuttings of *V. planifolia* from Belgium to make a new attempt to introduce vanilla culture into Java. About two dozen Belgian plants are taken via Leiden by Pierot to Indonesia, constituting the second introduction of the species [48,49].
September 1841	Morren attends the third meeting of Italian Scientists in Firenze exposing his results on *Vanilla* pollination in Italy [66].
10 November 1841	*Vanilla* production is suggested as an industry with a promising future in Réunion in an article published in Feuille Hebdomadaire signed by the initials L.B. The letter requests that the Comité d’Agriculture work on this [89].
6 July 1842	The members of the Comité d’Agriculture are invited to the reading of a memoir by M. Dupuy, naval pharmacist of the first class, in Guadeloupe, on *Vanilla* culture. Including indications on a new procedure that has succeeded in France and the West Indies in making this, previously sterile, plant productive.
12 September 1842	Mr. Montmerqué (as Montmerquier) arrives at La Réunion on the ship l’Union.
26 June 1843	Professor Roberto de Visiani offers a letter on the fruiting of *V. planifolia* in the Botanical Garden of Padua in which he clearly states Morren’s and Neumann’s experiments as inspiration for his own [53].
30 August 1843	Edmond’s practical way to pollinate *Vanilla* is published in the article Fécondation artificielle des fleurs de vanillier-aromatique in the Feuille Hebdomadaire [90].
16 December 1844	Teijsmann confirms that the *V. planifolia* plants sent to Java are growing well, have now bloomed [51].
1846	*Vanilla* exports from la Réunion are recorded for the first time, amounting to 38 kg.
1846–1857	Morren is named Knight of the North Star of Sweden and Norway (25 June 1846). Named Chevalier de l’Ordre de Léopold, King of Belgium (8 September 1846). Named Chevalier de Notre Ordre de la Couronne de Chêne by William III King of the Netherlands, Prince of Orange-Nassau and Grand duke of Luxembourg (2 May 1849). Named Ridder af Dannebrogordenen (Knight of the Order of the Danes) by his Majesty the Frederick VII King of Denmark (1 April 1850). Named Knight of the Order of the Württemberg Crown (Orden der Württembergischen Krone) (16 January 1852). Named Cavaleiro da Real Ordem Militar Portuguesa de Nosso Senhor Jesus Christo (Knight of the Military Order of Christ) by King Pedro V, regent of the Kingdom of Portugal (7 September 1854). Conferred the great gold medal by Prince Volkonsky, minister of the house of the Royal Imperial Majesty of the Russian Empire (29 July 1857).
20 December 1848	Emancipation. The decree abolishing slavery is applied in Réunion island.
1850	Teijsmann points out that news has finally arrived on how to fertilize *V. planifolia* and this can be applied to expand its culture in Java (Indonesia).
8 December 1853	Mézières Lépervanche first mentions Edmond Albius as the inventor of the procedure to pollinated *Vanilla* on Réunion in a letter to the governor. Edmond was sent to Mr. Patu de Rosemond, Mr. Floris, Mr. Desbassayns, and Mr. Vinet [86].
1856	De Vriese credits Morren as first discoverer of the *Vanilla* pollination, pointing out his method produced fruits in Liege, Paris, Kew, Padua, Berlin, and many other European gardens [49].
30 April 1860	A letter by Perrottet is published in the Moniteur officiel of Pondichéry, in the letter he asserts having brought news of Neumann’s pollination of *V. planifolia* to Patu de Rosemond, Bellier-Beaumont and Lépervanche on Réunion in 1839 [76].
17 February 1861	Bellier-Beaumont sends a letter to the judge stating Edmond Albius discovered how to pollinate *Vanilla* somewhere between 1840 and 1842, as would be evidenced in a letter addressed to Mr. Montmarqué [86].
1862	Edmond Albius’ name is first appears published in relation to the pollination of *Vanilla* in works by Maillard [72] and Focard [77], and introduced by the latter as an ingenious symbol of creole culture and resistance.

## Data Availability

Any additional information can be requested directly from the author.

## References

[1] Food and Agriculture Organization of the United Nations (2022). FAOSTAT Database. https://www.fao.org/faostat/en/#data/QCL.

[2] Watteyn C., Reubens B., Azofeifa Bolaños J.B., Solano Campos F., Pérez Silva A., Karremans A.P., Muys B. (2023). Cultivation potential of *Vanilla* crop wild relatives in two contrasting land use systems. Eur. J. Agron..

[3] Bramel P., Frey F. (2021). Global Strategy for the Conservation and Use of Vanilla Genetic Resources.

[4] Bory S., Grisoni M., Duval M.F., Besse P. (2008). Biodiversity and preservation of *Vanilla*: Present state of knowledge. Genet. Resour. Crop Evol..

[5] Karremans A.P., Chinchilla I.F., Rojas-Alvarado G., Cedeño-Fonseca M., Damián A., Léotard G. (2020). A reappraisal of Neotropical *Vanilla*. With a note on taxonomic inflation and the importance of alpha taxonomy in biological studies. Lankesteriana.

[6] Villanueva-Viramontes S., Hernández-Apolinar M., Carnevali Fernández-Concha G., Dorantes-Euán A., Dzib G.R., Martínez- Castillo J. (2017). Wild *Vanilla planifolia* and its relatives in the Mexican Yucatan Peninsula: Systematic analyses with ISSR and ITS. Bot. Sci..

[7] Cameron K.M. (2011). Vanilla Orchids: Natural History and Cultivation.

[8] Watteyn C., Scaccabarozzi D., Muys B., Reubens B., Ackerman J.D., Fernández Otárola M., Guizar Amador M.F., Karremans A.P. (2023). Sweet as *Vanilla hartii*: Evidence for nectar rewards in *Vanilla* (Orchidaceae) flowers. Flora.

[9] Lubinsky P., Van Dam M., Van Dam A. (2006). Pollination of *Vanilla* and evolution in Orchidaceae. Lindleyana.

[10] van Dam A.R., Householder J.E., Lubinsky P. (2010). *Vanilla bicolor* Lindl. (Orchidaceae) from the Peruvian Amazon: Auto-fertilization in *Vanilla* and notes on floral phenology. Genet. Resour. Crop Evol..

[11] Pansarin E.R., Pansarin L.M. (2014). Floral biology of two Vanilloideae (Orchidaceae) primarily adapted to pollination by euglossine bees. Plant Biol..

[12] Gigant R.L., De Bruyn A., M’sa T., Viscardi G., Gigord L., Gauvin-Bialecki A., Pailler T., Humeau L., Grisoni M., Besse P. (2016). Combining pollination ecology and fine-scale spatial genetic structure analysis to unravel the reproductive strategy of an insular threatened orchid. S. Afr. J. Bot..

[13] Watteyn C., Scaccabarozzi D., Muys B., Van der Schueren N., Van Meerbeek K., Guizar Amador M.F., Ackerman J.D., Cedeño Fonseca M.V., Chinchilla Alvarado I.F., Reubens B. (2022). Trick or treat? Pollinator attraction in *Vanilla pompona* (Orchidaceae). Biotropica.

[14] Ackerman J.D. (1983). Specificity and mutual dependency of the orchid-euglossine bee interaction. Biol. J. Linn. Soc..

[15] Householder E., Janovec J., Mozambite A.B., Maceda J.H., Wells J., Valega R., Maruenda H., Christenson E. (2010). Diversity, natural history, and conservation of *Vanilla* (Orchidaceae) in Amazonian wetlands of Madre de Dios, Peru. J. Bot. Res. Inst. Texas.

[16] Soto Arenas M.A., Dressler R.L. (2010). A revision of the Mexican and Central American species of Vanilla Plumier ex Miller with a characterization of their ITS region of the nuclear ribosomal DNA. Lankesteriana.

[17] Anjos A.M., Barbarena F.F.V.A., Pigozzo C.M. (2016). Biologia reprodutiva de *Vanilla bahiana* Hoehne (Orchidaceae). Orquidário.

[18] Lubinsky P., Romero-Gonzalez G.A., Heredia S.M., Zabel S., Havkin-Frenkel D., Belanger F.C. (2019). Origins and patterns of Vanilla cultivation in tropical America (1500–1900): No support for independent domestication of vanilla in South America. Handbook of Vanilla Science and Technology.

[19] Lubinsky P., Cameron K.M., Molina M.C., Wong M., Lepers-Andrzejewski S., Gómez-Pompa A., Kim S.C. (2008). Neotropical roots of a Polynesian spice: The hybrid origin of Tahitian vanilla, *Vanilla* × *tahitensis* (Orchidaceae). Am. J. Bot..

[20] Arditti J. (2021). Discoverers of *Vanilla* hand pollination. Orchids (Bull. Amer. Orch. Soc.).

[21] Soto Arenas M.A., Pridgeon A.M., Cribb P.J., Chase M.W., Rasmussen F.N. (2003). Vanilla. Genera Orchidacearum: Orchidoideae (Part Two), Vanilloideae.

[22] Delteil A. (1884). La Vanille Sa Culture Et Sa Préparation.

[23] Bogarín D., Serracín Z., Samudio Z., Rincón R., Pupulin F. (2014). An updated checklist of the Orchidaceae of Panamá. Lankesteriana.

[24] Karremans A.P., Bogarín D., Fernández Otárola M., Sharma J., Watteyn C., Warner J., Rodríguez Herrera B., Chinchilla I.F., Carman E., Rojas Valerio E. (2022). First evidence for multimodal animal seed dispersal in orchids. Curr. Biol..

[25] Karremans A.P., Watteyn C., Scaccabarozzi D., Pérez-Escobar O.A., Bogarín D. (2023). Evolution of seed dispersal modes in the Orchidaceae: Has the *Vanilla* mystery been solved?. Horticulturae.

[26] Vargas-Acuña F. (2024). Personal communication.

[27] Quezada-Euán J.J.G., Guerrero-Herrera R.O., González-Ramírez R.M., MacFarlane D.W. (2024). Frequency and behavior of *Melipona* stingless bees and orchid bees (Hymenoptera: Apidae) in relation to floral characteristics of vanilla in the Yucatán region of Mexico. PLoS ONE.

[28] Dressler R.L. (1981). The Orchids: Natural History and Classification.

[29] Pemberton R.W., Wheeler G.S., Madeira P.T. (2023). Bee (Hymenoptera: Apidae) Pollination of *Vanilla planifolia* in Florida and Their Potential in Commercial Production. Fla. Entomol..

[30] Neiland M.R.M., Wilcock C.C. (1998). Fruit set, nectar reward, and rarity in the Orchidaceae. Am. J. Bot..

[31] Pemberton R.W. (2010). Biotic resource needs of specialist orchid pollinators. Bot. Rev..

[32] Dampier W. (1776). Voyages and Adventures of Captain William Dampier.

[33] Karremans A.P. (2023). Demystifying Orchid Pollination: Stories of Sex, Lies and Obsession.

[34] Sprengel C.K. (1793). Das Entdeckte Geheimnis der Natur im Bau und in der Befruchtung der Blumen.

[35] Wachter J.K. (1801). Ueber die merkwürdige Ortsveränderung der Antheren, und Befruchtungsart der Linneischen Pflanzengeschlechter *Orchis*, *Ophrys*, *Serapias* und *Satyrium* nebst einiger botanischen Bemerkungen. Arch. Bot..

[36] Arditti J. (1984). An history of orchid hybridization, seed germination and tissue culture. Bot. J. Linn. Soc..

[37] Alisbury R.A. (1804). On the germination of the seeds of Orchidaceae. Trans. Linn. Soc. Lond..

[38] Brown R. (1833). On the organs and mode of fecundation in Orchideae and Asclepiadeae. Trans. Linn. Soc..

[39] Brongniart A. (1831). Observations sur le mode de fécondation des Orchidées et des Cistinées. Ann. Sci. Nat..

[40] Morren E. (1860). Notice sur Charles Morren. Annuaire de l’Académie Royale des Sciences, des Lettres et des Beaux-Arts de Belgique.

[41] Morren C. (1838). Notice sur la vanille indigène. Bull. Acad. Roy. Sci. Brux..

[42] Rolfe R.A. (1895). Vanillas of commerce. Kew Bull..

[43] Salisbury R.A. (1807). The Paradisus Londinensis or Coloured Figures of Plants Cultivated in the Vicinity of the Metropolis.

[44] Bauer F., Lindley J. (1834). Illustrations of Orchidaceous Plants.

[45] Andrews H.C. (1808). *Vanilla* *planifolia*. Bot. Repos..

[46] Cooke I.K.S. (1992). Whiteknights and the Marquis of Blandford. Gard. Hist..

[47] Aernouts R., Frison E. (1961). Claude Louis Somme. Heelmeester, dokter en botanicus (1772–1855). Sci. Hist..

[48] Morren C. (1850). Memorandum sur la Vanille, son histoire et sa culture. Bull. Acad. Roy. Sci. Belg..

[49] de Vriese W.H. (1856). Tuinbouw-Flora van Nederland en Zijne Overzeesche Bezittingen.

[50] van Gorkom K.W. (1884). De Oost-Indische Cultures, in Betrekking tot Handel en Nijverheid.

[51] Teijsmann J.E. (1850). Lands Plantentuin te Buitenzorg. Natuurk. Tijdschr. Ned.-Indie.

[52] Beaujean J. (2002). Petite histoire de l’introduction et de la fructification du vanillier (*Vanilla planifolia* Jacks. ex Andrews) au jardin botanique de l’Université de Liège et à l’île de la Réunion. Nat. Mosana.

[53] de Visiani R. (1844). Del Metodo e Delle Avvertenze che si Usano nell’Orto Botanico di Padova per la Cultura e la Fruttificazione Della Vaniglia.

[54] (1840). Observation sur l’odeur de fruit du *Leptotes bicolor*. Ann. Soc. Hort. Paris.

[55] Morren C. (1837). Note sur le première fructification du vanillier en Europe. Ann. Soc. Hort. Paris.

[56] Morren C. (1839). On the Production of *Vanilla* in Europe. Ann. Nat. Hist..

[57] Berlèse L., Poiteau P.A. (1837). Rapport fait á la Societé royale d’horticulture de Paris, le 5 avril 1837, sur la cinquante-sixième exposition publique de la Société royale d’agriculture et de botanique de Gand, en mars dernier. Ann. Soc. Hort. Paris.

[58] Poiteau P.A., Berlèse L. (1837). Second rapport sur la première exposition horticole faite dans le Casino, à Gand, le 10 mars 1837. Ann. Soc. Hort. Paris.

[59] Camuzet J.B. (1837). Tournée horticole faite en Belgique, en septembre dernier. Hort. Belge.

[60] Soulange-Bodin E. (1838). Compte rendu des travaux de la Société Royale D’Horticulture de Paris. Ann. Soc. Hort. Paris.

[61] Delteil A. (1874). Étude sure La Vanille.

[62] Neumann J. (1838). Vanille. Ann. Fl. Pomone.

[63] Neumann J. (1839). Vanille a feuiles planes, *Vanilla planifolia*. Hort. Univers..

[64] Poiteau P.A. (1838). Note sur la Vanille. Ann. Soc. Hort. Paris.

[65] de Thury H. (1840). Note sur le prix de la Vanille. Ann. Soc. Hort. Paris.

[66] Savi P. (1841). Atti Della Terza Riunione Degli Scienziati Italiani.

[67] Arditti J., Rao N., Nair H., Kull T., Arditti J., Wong S.K. (2009). Hand Pollination of *Vanilla*: How Many Discoverers. Orchid Biology, Reviews and Perspectives.

[68] Teijsmann J.E. (1858). Verslag ontent den staat vant’s Lands Plantentuin in Het Jaar 1850. Versllag van het Beheer en den Staat der Nederlandsche Besittingen en Kolonien in Oest and West Indie Enter kust von Guinea over 1850.

[69] Smits H.D.A. (1852). Kungsmatige Bevfruchting de Vanille te Buitenzorg. Natuurk. Tijdschr. Ned.-Indië.

[70] Raman A. (2014). Georges Guerrard Samuel Perrottet, a forgotten Swiss-French plant collector, experimental botanist and biologist in I dia. Curr. Sci. India.

[71] van Steenis C.G.G.J. (1950). Flora Malesiana.

[72] Maillard L. (1862). Notes sur l’île de la Réunion (Bourbon).

[73] Richard A. (1828). Monographie des Orchidées des Iles de France et de Bourbon. Mem. Soc. Hist. Nat. Paris.

[74] Richard A. (1841). Monographie des Orchidées Recueillies dans la Chaine des Nil-gherries (Indes-Orientales) par M. Perrottet.

[75] Delessert A. (1843). Souvenirs d’un voyage dans l’Inde Execute de 1834 à 1839.

[76] Perrottet G.S. (1860). Introduction du Vanillier á la Réunion. Pondichéry.

[77] Focard V. (1862). Introduction et fécondation du vanillier à l’île Bourbon. Bull. Soc. Sci. Arts Reun..

[78] Focard V., Roussin A. (1863). Le vanillier, la vanille. Album de l’île de la Réunion.

[79] Aimar P. (2010). Vanille: La route Bourbon.

[80] Jennings E. (2025). Vanilla, a World History.

[81] Wood C.A. (1922). Louis Daniel Beauperthuy. Ann. Med. Hist..

[82] Sakula A. (1986). Louis Daniel Beauperthuy: Pioneer in Yellow Fever and Leprosy Research. J. R. Coll. Physicians Lond..

[83] Traviezo Valles L.E. (2020). Luis Daniel Beauperthuy, filántropo y precursor de la entomología médica. Saber.

[84] (1914). La production de la vanille dans les colonies Françaises. Bull. Off. Colon..

[85] Jennings E. (2023). The Enslaved Teen Who Cracked Vanilla’s Secret.

[86] Chabin M. (1981). Edmond Albius et la découverte de la fécondation artificielle de la vanille: La correspondance entre Bellier-Beaumont et Volsy-Focard. Arch. Bourbon.

[87] Lépervanche M.A. (1840). Notes et observations sur le caféier et sur la nécessité d’en renouveler la semence a Bourbon. Ann. Com. Agric..

[88] Lépervanche M.A. (1841). De la culture du café a Salazie. Ann. Com. Agric..

[89] (1841). Anonymous [L.B. (Saint-Louis, 1 nov 1841)]. Industrie agricole de la vanille. Feuille Hebd..

[90] (1843). Anonymous [L.P.]. Fécondation artificielle des fleurs de vanillier-aromatique. Feuille Hebd..

[91] Ecott T. (2004). Vanilla: Travels in Search of the Ice Cream Orchid.

[92] Focard V., Focard V. (1863). Introduction et fécondation de la vanille à Bourbon. Dix-Huit Mois de République à L’ile Bourbon.

[93] Fouché J.-G., Jouve L. (1999). *Vanilla planifolia*: History, botany and culture in Réunion island. Agronomie.

[94] Lucas R., Odoux G., Grisoni M. (2011). Vanilla’s debt to Reunion island. Vanilla. Medicinal and Aromatic Plants—Industrial Profiles.

[95] Lucas R. (1990). La Réunion ile de Vanille.

[96] Robbirt K., Roberts D.L., Hutchings M.J., Davy A.J. (2014). Potential disruption of pollination in a sexually deceptive orchid by climatic change. Curr. Biol..

[97] Tsiftsis S., Djordjević V. (2020). Modelling sexually deceptive orchid species distributions under future climates: The importance of plant–pollinator interactions. Sci. Rep..

[98] Dafni A., Firmage D. (2000). Pollen viability and longevity: Practical, ecological and evolutionary implications. Plant Syst. Evol..

[99] Pacini E., Dolferus R. (2019). Pollen Developmental Arrest: Maintaining Pollen Fertility in a World With a Changing Climate. Front. Plant Sci..

[100] Pacini E., Hesse M. (2002). Types of pollen dispersal units in orchids. and their consequences for germination and fertilization. Ann. Bot..

[101] Pacini E., Kull T., Arditti J., Wong S.K. (2009). Orchids pollen dispersal units and reproductive consequences. Orchid Biology, Reviews and Perspectives.

[102] Brownell R.J., Havkin-Frenkel D., Belanger F.C. (2019). Fair Trade—The Future of Vanilla?. Handbook of Vanilla Science and Technology.

[103] Goettsch B., Urquiza-Haas T., Koleff P., Acevedo Gasman F., Aguilar-Meléndez A., Alavez V., Alejandre-Iturbide G., Aragón Cuevas F., Azurdia Pérez C., Carr J.A. (2021). Extinction risk of Mesoamerican crop wild relatives. Plants People Planet.

[104] Armenta-Montero S., Menchaca-García R., Pérez-Silva A., Velázquez-Rosas N. (2022). Changes in the Potential Distribution of *Vanilla planifolia* Andrews under Different Climate Change Projections in Mexico. Sustainability.

[105] Flanagan N.S., Mosquera-Espinosa A.T. (2016). An integrated strategy for the conservation and sustainable use of native *Vanilla* species in Colombia. Lankesteriana.

[106] Hänke H. (2024). Living Income Reference Price for Vanilla from Madagascar.

[107] Watteyn C. (2024). Personal communication.

[108] Come B. (2024). Personal communication.

[109] Watteyn C., Fremout T., Karremans A.P., Huarcaya R.P., Azofeifa Bolanos J.B., Reubens B., Muys B. (2020). *Vanilla* distribution modeling for conservation and sustainable cultivation in a joint land sparing/sharing concept. Ecosphere.

[110] Li J., Demesyeux L., Brym M., Chambers A.H. (2020). Development of species-specific molecular markers in *Vanilla* for seedling selection of hybrids. Mol. Biol. Rep..

[111] Grisoni M., Nany F. (2021). The beautiful hills: Half a century of *Vanilla* (*Vanilla planifolia* Jacks. ex Andrews) breeding in Madagascar. Genet. Resour. Crop Evol..

[112] Besse P., Da Silva D., Bory S., Grisoni M., Le Bellec F., Duval M.F. (2004). RAPD genetic diversity in cultivated vanilla: *Vanilla planifolia*, and relationships with *V. tahitensis* and *V. pompona*. Plant Sci..

[113] Bory S., Da Silva D., Risterucci A.M., Grisoni M., Besse P., Duval M.F. (2008). Development of microsatellite markers in cultivated vanilla: Polymorphism and transferability to other vanilla species. Sci. Hortic..

[114] Ramos-Castella A.L., Iglesias-Andreu L.G., Martínez-Castillo J., Ortiz-García M., Andueza-Noh R.H., Octavio-Aguilar P., Luna-Rodríguez M. (2016). Evaluation of molecular variability in germplasm of vanilla (*Vanilla planifolia* G. Jackson in Andrews) in Southeast Mexico: Implications for genetic improvement and conservation. Plant Genet. Res..

[115] Chambers A., Cibrián-Jaramillo A., Karremans A.P., Martinez D.M., Hernandez-Hernandez J., Brym M., Resende M.F., Moloney R., Sierra S.N., Hasing T. (2021). Genotyping-By-Sequencing diversity analysis of international Vanilla collections uncovers hidden diversity and enables plant improvement. Plant Sci..

[116] Ellestad P., Farrera M.A.P., Forest F., Buerki S. (2022). Uncovering haplotype diversity in cultivated Mexican vanilla species. Am. J. Bot..

[117] Soto-Arenas M.A. (1999). Filogeografía y Recursos Genéticos de las Vainillas de México.

[118] Roux-Cuvelier M., Grisoni M., Odoux G., Grisoni M. (2011). Conservation and movement of *Vanilla* germplasm. Vanilla. Medicinal and Aromatic Plants—Industrial Profiles.

[119] Lozano Rodríguez M.A., Rodríguez M.L., Canche J.M.P., García R.A.M., Cabrera C.R.C. (2022). Visit frequency of Euglossine bees (Hymenoptera: Apidae) to mature fruits of *Vanilla planifolia* (Orchidaceae). Acta Bot. Mex..

[120] Wong S., Kaur J., Kumar P., Karremans A.P., Sharma J. (2024). Distinct orchid mycorrhizal fungal communities among co-occurring *Vanilla* species in Costa Rica: Root substrate and population-based segregation. Mycorrhiza.

[121] Serna-Sánchez A., Fuchs E.J., Bogarín D., Karremans A.P. (2024). Wild Vanilla populations exhibit low genetic diversity and contrasting genetic structure between two species. Centro de Investigación Jardín Botánico Lankester, Universidad de Costa Rica, Cartago, Apartado 302-7050, Costa Rica. Sci. Rep..

[122] Watteyn C., Fremout T., Karremans A.P., van Meerbeek K., Lipinska M., Janssens S., de Backer S., Muys B. (2024). Climate change effects on Neotropical *Vanilla* species (Orchidaceae) and their pollinators: A guidance to integral conservation efforts in the Americas. Department of Earth and Environmental Sciences, KU Leuven, Celestijnenlaan 200E, Box 2411, 3001, Leuven, Belgium. Plants.

